# *De novo* assembly and sex-specific transcriptome profiling in the sand fly *Phlebotomus perniciosus* (Diptera, Phlebotominae), a major Old World vector of *Leishmania infantum*

**DOI:** 10.1186/s12864-015-2088-x

**Published:** 2015-10-23

**Authors:** V. Petrella, S. Aceto, F. Musacchia, V. Colonna, M. Robinson, V. Benes, G. Cicotti, G. Bongiorno, L. Gradoni, P. Volf, M. Salvemini

**Affiliations:** Department of Biology, University of Naples Federico II, Naples, Italy; Stazione Zoologica “Anton Dohrn”, Naples, Italy; National Research Council, Institute of Genetics and Biophysics, Naples, Italy; Institute of Molecular Life Science, University of Zurich, Zurich, Switzerland; SIB—Swiss Institute of Bioinformatics, University of Zurich, Zurich, Switzerland; Genomics Core Facility, EMBL, Heidelberg, Germany; Institute for High Performance Computing and Networking, ICAR-CNR, Naples, Italy; Department of Infectious, Parasitic and Immunomediated Diseases, Istituto Superiore di Sanità, Rome, Italy; Department of Parasitology, Charles University, Prague, Czech Republic

**Keywords:** Nematocera, Sand fly, Transcriptome *de novo* assembly, RNA-seq, Differential expression analysis, Sex-specific gene expression, Sex-biased genes

## Abstract

**Background:**

The phlebotomine sand fly *Phlebotomus perniciosus* (Diptera: Psychodidae, Phlebotominae) is a major Old World vector of the protozoan *Leishmania infantum*, the etiological agent of visceral and cutaneous leishmaniases in humans and dogs, a worldwide re-emerging diseases of great public health concern, affecting 101 countries. Despite the growing interest in the study of this sand fly species in the last years, the development of genomic resources has been limited so far. To increase the available sequence data for *P. perniciosus* and to start studying the molecular basis of the sexual differentiation in sand flies, we performed whole transcriptome Illumina RNA sequencing (RNA-seq) of adult males and females and *de novo* transcriptome assembly.

**Results:**

We assembled 55,393 high quality transcripts, of which 29,292 were unique, starting from adult whole body male and female pools. 11,736 transcripts had at least one functional annotation, including full-length low abundance salivary transcripts, 981 transcripts were classified as putative long non-coding RNAs and 244 transcripts encoded for putative novel proteins specific of the Phlebotominae sub-family. Differential expression analysis identified 8590 transcripts significantly biased between sexes. Among them, some show relaxation of selective constraints when compared to their orthologs of the New World sand fly species *Lutzomyia longipalpi*s.

**Conclusions:**

In this paper, we present a comprehensive transcriptome resource for the sand fly species *P. perniciosus* built from short-read RNA-seq and we provide insights into sex-specific gene expression at adult stage. Our analysis represents a first step towards the identification of sex-specific genes and pathways and a foundation for forthcoming investigations into this important vector species, including the study of the evolution of sex-biased genes and of the sexual differentiation in phlebotomine sand flies.

**Electronic supplementary material:**

The online version of this article (doi:10.1186/s12864-015-2088-x) contains supplementary material, which is available to authorized users.

## Background

In the Old World, the sand fly *Phlebotomus* (*Larroussius*) *perniciosus* (Diptera: Psychodidae, Phlebotominae) is one of the major vectors of *Leishmania infantum* (Kinetoplastida: Trypanosomatidae), the parasitic protozoan that causes visceral and cutaneous leishmaniases in humans and canine reservoir hosts [1–3]. *P. perniciosus* is also a vector for various known and emerging arboviruses considered relevant from a public health perspective [4, 5]. Described for the first time in Malta by Newstead in 1911, *P. perniciosus* is widely distributed in the western Mediterranean Basin, from Morocco to Libya in North Africa and from Portugal to Croatia in Europe. One of the most important endemic foci of canine and human visceral leishmaniases of this area is located in Italy, in the Campania region [6]. Leishmaniases are diseases of great public health concern affecting 101 countries around the world with an estimated incidence of 0.9–1.6 million new cases each year [7]. Due to climate and social changes, leishmaniases are becoming a worldwide re-emerging public health problem, with an expanding endemicity and more than 300 million people estimated at risk of transmission world-wide [8]. Despite their importance as disease vectors, sand flies remain understudied relative to mosquitoes and other important vectors and relatively little is known about their biology, their feeding and reproductive behaviour under natural conditions, and about their genetics [9, 10].

In the recent years, the advent of the next generation sequencing technology and bioinformatics applied to the study of insects have paved the way for the fast identification and characterization of genes involved in relevant biological processes [11–13]. Nowadays, the number of insect genomes and transcriptomes available is increasing exponentially in public databases [14] (http://www.1kite.org). This allows for easier and faster comparative studies between related species.

To date two unpublished genome sequencing projects of the New World species *Lutzomyia longipalpis* (Dillon et al., unpub. res.; https://www.vectorbase.org/organisms/lutzomyia-longipalpis) and of the Old World species *P. papatasi* (McDowell et al. unpub. res.; https://www.vectorbase.org/organisms/phlebotomus-papatasi) and a few transcriptomics studies are available for sand fly species. Most transcriptomics studies, performed with EST sequencing, have focused on sand fly salivary glands and the role of saliva in pathogen-vector-host interactions [15–20], on sand fly-*Leishmania* interactions [21–23] and on the analysis of sand fly specific tissues [24]. Only three studies have performed a global sand fly transcriptome analysis, the first two using EST sequencing technology [25, 26] and the third by 454 pyrosequencing on wild individuals of *L. longipalpis* [27]. In addition, only two studies are available about the salivary gland and midgut transcriptome of *P. perniciosus* [28, 29].

In the present paper, we report the *de novo* transcriptome assembly and the differential expression analysis of adult males and females of the sand fly *P. perniciosus*. This analysis led to the identification of about 8600 sex-biased genes and represents the first next generation sequencing study with Illumina technology for a sand fly species. The data set constitutes a relevant resource for future genome annotation projects for *P. perniciosus* and for evolutionary comparative studies with other sand fly species and other blood-sucking Nematocera species, such as mosquitoes (Culicidae), biting midges (Ceratopogonidae) and black flies (Simuliidae). In particular, our data can be useful for the study of genes involved in the control of sex-specific traits such as host-parasite interaction, reproductive biology, somatic sexual development and sex determination, accelerating the discovery of potential targets for vector-based control strategies against leishmaniasis and other arthropod-borne diseases.

## Results and discussion

### Sequencing and *de novo* assembly of the *P. perniciosus* transcriptome

**Fig. 1:**
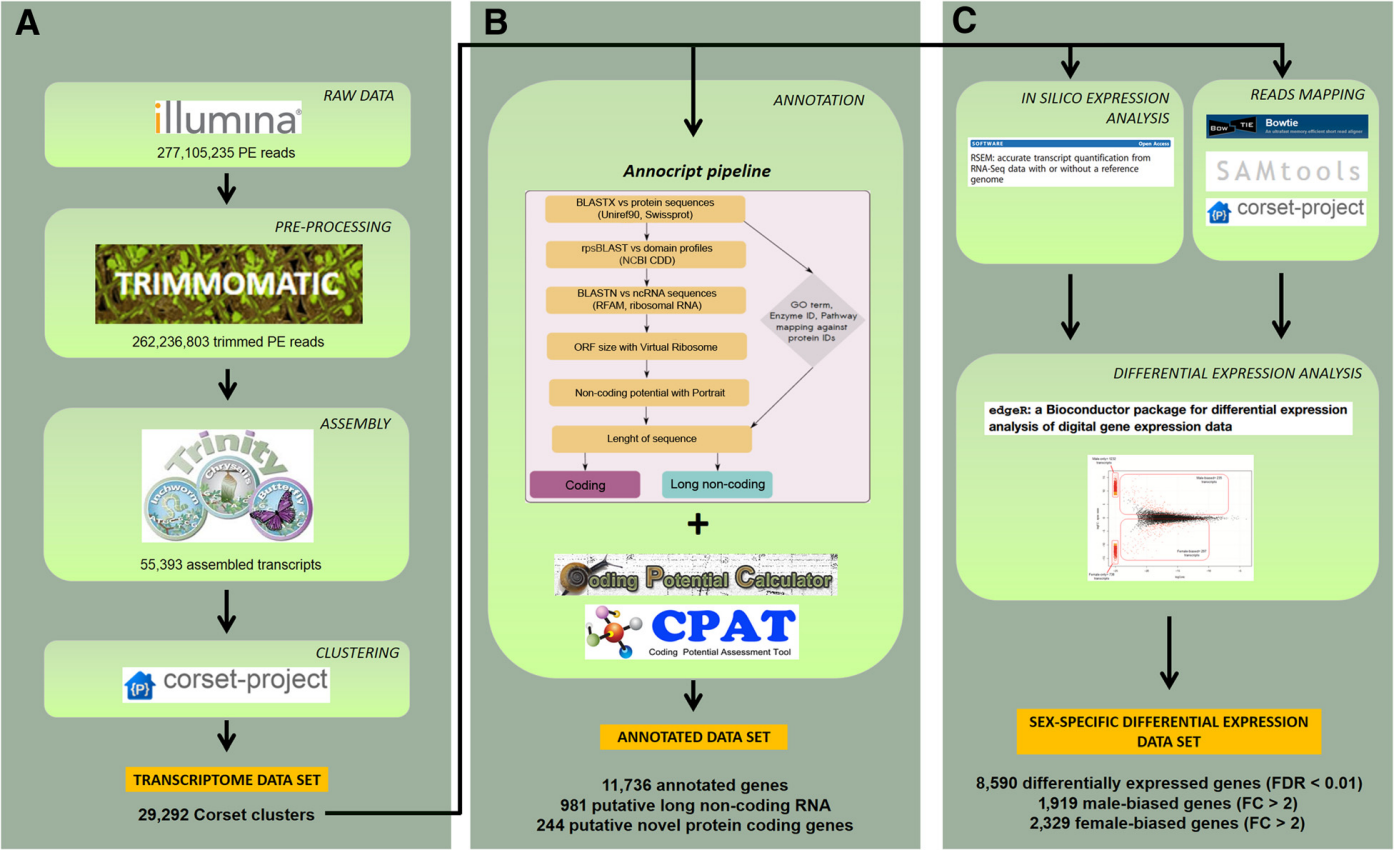
Analysis workflow. Flowchart illustrating the main steps of the bioinformatics pipeline: **a** assembly, (**b**) annotation and (**c**) differential expression analysis. FDR = false discovery rate, FC = fold change

To generate a comprehensive adult reference transcriptome of *P. perniciosus* and to investigate sex-biased gene expression levels at adult stage, we produced six Illumina RNA-seq libraries, with an average insert size of 130 bp, from three pools of adult sand flies for each sex. Illumina deep sequencing of the libraries yielded about 280 million paired-end reads that were combined, quality filtered, and *de novo* assembled using the Trinity software [30, 31]. We obtained 55,393 assembled transcripts with a minimum length of 201 bp and a N50 value of 2376 bp. The assembled transcripts were grouped in gene clusters (referred to henceforth as “Corset clusters”) using Corset [32] and, after discarding clusters with less than 10 mapped reads, we obtained 29,292 Corset clusters of which 20,667 (70.5 %) contain only one transcript isoform, while the remaining 8625 clusters (29.5 %) have 2–25 transcript isoforms (median = 2, mean = 3, standard deviation = 1.8). We selected the longest transcript of multiple isoform Corset clusters as representative of the cluster, obtaining a final data set of 29,292 unique transcripts that we named the “PERNI” data set. Sequencing and assembly statistics are summarized in Table 1 and an overview of the assembly, the annotation and the differential expression analysis workflow is presented in Fig. 1. Sequencing data were deposited into the Short Read Archive (SRA) of the NCBI (Accession Number: SRP059770).

**Table 1:** Summary of sequencing and assembly statistics

Total sequenced reads	277,105,235
Trimmomatic filtered reads	262,236,803
Total assembled bases (bp)	69,451,297
Trinity assembled transcripts	55,393
Corset clusters	29,292
Median transcript lenght (bp)	645
Average transcript lenght (bp)	1253.79
Transcript N50 (bp)	2376
Shortest transcript lenght (bp)	201
Longest transcript lenght (bp)	25,399
Trinity transcripts > 1Kb	21,529
Trinity transcripts > 2Kb	11,192

### Transcriptome quality assessment

We validated the PERNI data set for sequence completeness using three different approaches. First, we applied the ortholog hit ratio metric (OHR) [33], which provides an estimate of the length of the assembled transcripts using their coding regions. OHR compares the length of the coding region between a newly assembled transcript and its ortholog of a reference transcriptome. If the two have the same length, then the reconstructed transcript is likely to correspond to the full length real mRNA, including the upstream and downstream untranslated regions, and the OHR = 1. Values of OHR < 1 suggest incompleteness. We calculated OHR values of 2827 PERNI transcripts chosen to have best reciprocal TBLASTX hits (*E*-value: 1e-6; min. hit coverage 30 %) with the *D. melanogaster* transcriptome (BDGP v6.03). In ~70 % of the cases, hits cover at least 50 % of the corresponding *Drosophila* transcript coding regions (average and median OHR values of 0.65 and 0.63; Additional file 1: Table S1). Similar results were obtained on 3621 PERNI transcripts using as reference the transcriptome of the sand fly *P. papatasi*, available at the VectorBase web site (Phlebotomus-papatasi-Israel_TRANSCRIPTS_PpapI1.1), with average and median OHR values of 0.66 and 0.65, respectively (Additional file 1: Table S1). Our results are comparable to the efficiencies observed in other *de novo* assembled transcriptomes of insects [34–36].

Next, we compared the PERNI data set with 4516 *P. perniciosus* nucleotide sequences available in the NCBI GenBank database (accession date 2015.01.15), mostly ESTs from a midgut transcriptome analysis [29]. The 4516 available sequences of *P. perniciosus* were clustered using CD-HIT [37] with default parameters to obtain 2869 unique sequences with a N50 value of 798 bp to be used for bidirectional BLASTN analysis (cut-off *E*-value of 1e-20) with the PERNI data set. We observed that while only 2 % of the PERNI data set matched available *P. perniciosus* sequences, 77 % (2219 out of 2869) of the *P. perniciosus* sequences available in GenBank matched transcripts in the PERNI data set. Furthermore, we observed that 1470 out of these 2219 *P. perniciosus* sequences available in GenBank are shorter than their corresponding transcripts in the PERNI data set. Besides validating it, these results indicate that the *de novo* assembly presented here improves the coverage and the average transcript length of *P. perniciosus*.

Finally, to test the ability to detect transcripts with low expression, we searched for three low-abundance salivary transcripts identified in other closely-relative sand fly species that are not yet identified in *P. perniciosus* [28] (Nikola Polanska pers. comm.): the *hyaluronidase*, *pyrophosphatase* and *adenosine deaminase* genes. We performed a BLASTP search of the protein sequences (*P. orientalis hyaluronidase* GenBank acc. num.: AGT96452.1; *P. argentipes pyrophosphatase* GenBank acc. num.: ABA12155.1; *P. duboscqi adenosine deaminase* GenBank acc. num.: ABI20162.1) against the PERNI data set. Despite the PERNI data set deriving from whole body male and female adults, for each of the three low-abundance salivary-specific genes, we identified a full *P. perniciosus* assembled orthologous transcript with a complete ORF (*hyaluronidase* gene: 1800 bp long transcript, 470 aa long putative protein, GenBank acc. num.: KT160228; *pyrophosphatase* gene: 2795 bp long transcript, 475 aa long putative protein, GenBank acc. num.: KT160227; *adenosine deaminase* gene: 2180 bp long transcript, 508 aa long putative protein, GenBank acc. num.: KT160229). In Additional file 2: Figure S1 we reported the multiple alignment of these three putative proteins of *P. perniciosus* with orthologous proteins present in GenBank.

Overall, these results indicate that we produced a high quality *de novo* assembly of *P. perniciosus* transcriptome that matches and improves existing information and includes transcripts of genes with low expression.

### Functional annotation

#### Prediction of protein coding transcripts

**Fig. 2:**
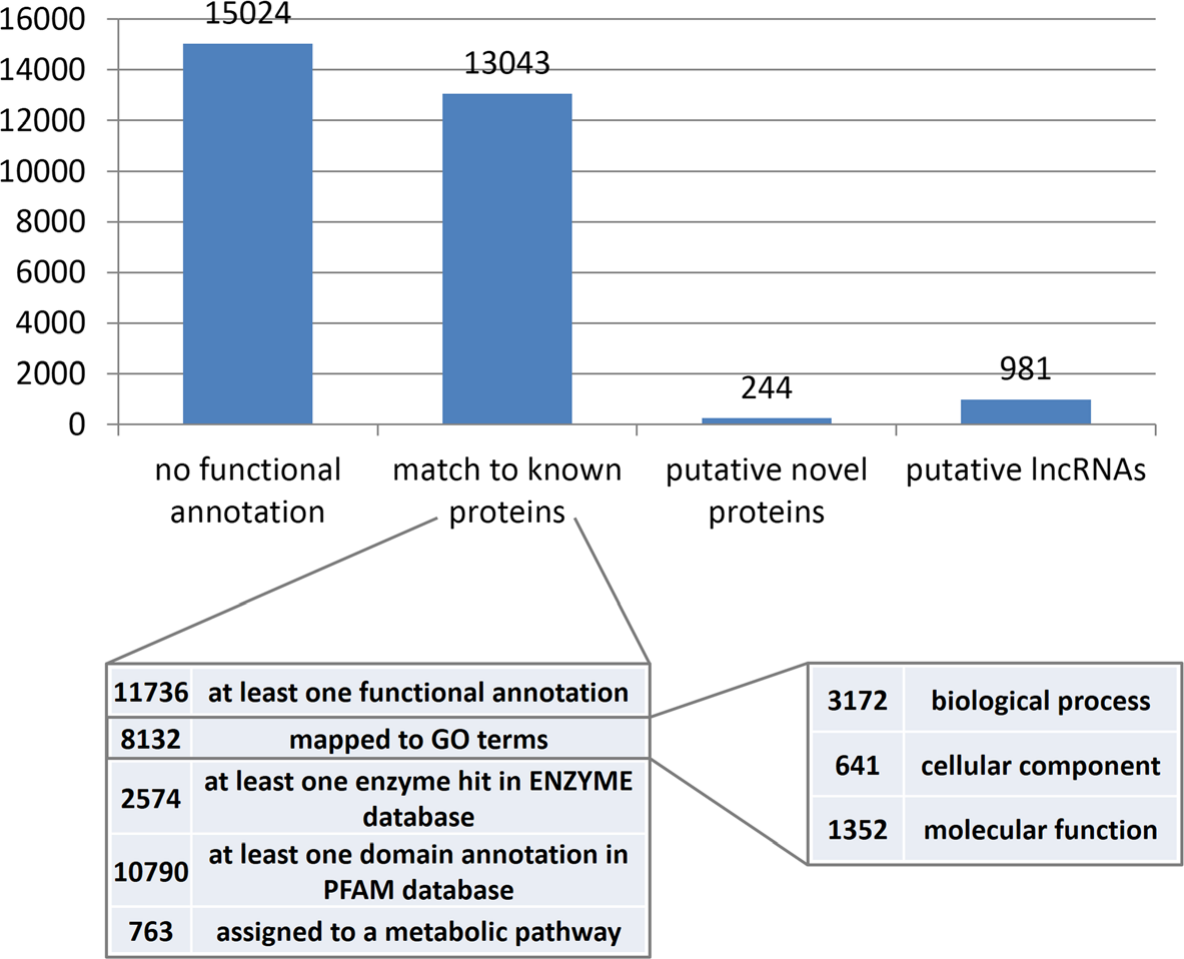
Summary of the functional annotation results. The bar plot represents the number of transcripts of the PERNI data set for each annotation category

We assigned functional annotation to the PERNI data set using the Annocript pipeline [38] with default parameters and using the UniprotKB (2014–11 version) as reference. The assembled and annotated PERNI data set is freely accessible at http://pernibase.evosexdevo.eu. We found that about 13,000 PERNI transcripts (44.5 % of total PERNI data set) had significant similarity (*E*-value < 1e-5) to proteins in the UniprotKB database. Among them, about twelve thousands PERNI transcripts (40 %) have at least one functional annotation (Fig. 2). In particular, (i) 8132 transcripts (27.7 %) mapped to GO terms; (ii) 2574 (8.8 %) had at least one enzyme hit in the ENZYME database (http://enzyme.expasy.org/); (iii) 10,790 (36.8 %) had at least one domain annotation in PFAM database (http://pfam.sanger.ac.uk/); (iv) 763 (2.6 %) were assigned to an UniPathway metabolic pathway (www.grenoble.prabi.fr/obiwarehouse/unipathway) (Additional file 3: Figure S2). Similarly to other recently released transcriptomes of dipteran insects [36, 39, 40], among the 8132 PERNI transcripts with an assigned GO term we observed 5165 GO functional categories belonging to biological process (BP), cellular component (CC) and molecular function (MF), suggesting that transcripts in the assembly presented here cover a wide spectrum of biological processes (Additional file 4: Table S2).

**Fig. 3:**
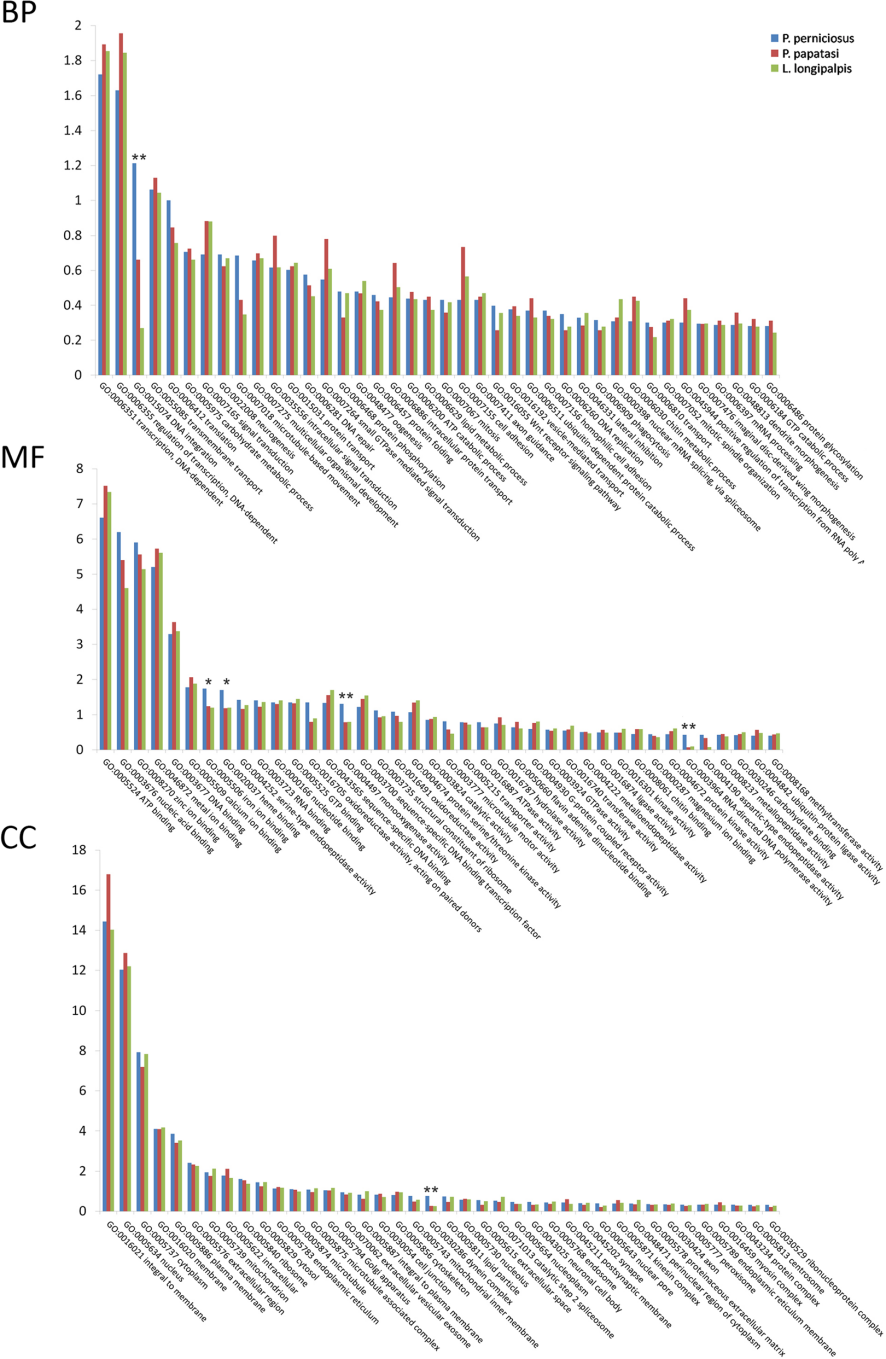
GO term distribution of the annotated transcripts of the PERNI data set compared with those of *P. papatasi* and *L. longipalpis*. GO categories are shown within the divisions of Biological Process (BP), Molecular Function (MF), and Cellular Component (CC). Column heights reflect the percentage of annotated transcripts in each assembly that mapped to a given GO term. Asterisks indicate the statistical significance of the differences observed in the pair-wise comparison between *P. perniciosus* and *P. papatasi* and between *P. perniciosus* and *L. longipalpis* (* = *P* < 0.05. ** = *P* < 0.01)

We then compared the GO terms distribution of the PERNI data set with that of the two available sand fly transcriptome data sets, the New World species *Lutzomyia longipalpis* and the Old World species *P. papatasi* [25, 26]. To prevent bias from different annotations methods the *L. longipalpis* and the *P. papatasi* data sets were re-annotated using the Annocript pipeline with default parameters, obtaining results in general agreement with previous annotations of the two sand fly transcriptomes [25, 26]. The overall distribution of the GO terms are similar for the three sand fly species (Fig. 3). In particular, in the MF category the “ATP binding” and the “nucleic acid binding” terms are the most abundant (7.15 % +/− 0.48 % and 5.40 % +/− 0.80 %, respectively) in all the species. In the BP category, the two most represented terms are “transcription” (1.82 % +/− 0.09 %) and “regulation of transcription” (1.81 % +/− 0.17 %). In the CC category, the two most abundant terms are “integral to membrane” (15.09 % +/− 1.49 %) and “nucleus” (12.37 % +/− 0.44 %). One interesting exception in the comparison of the three sand fly transcriptomes was a significant enrichment in the PERNI data set with respect to the BP term “DNA integration” (GO:0015074) and of the MF term “RNA-directed DNA polymerase activity” (GO:0003964 ) (Fisher exact test and FDR *p*-value correction, *p* < 0.01; Additional file 5: Table S3). We performed a more specific search of transposable elements in the PERNI data set using the protein-based RepeatMasker algorithm with default parameters (http://repeatmasker.org). We observed that about 5 % of the PERNI data set is constituted by transposable elements with the most abundant classes represented by LTR retrotrasposons of Gipsy and Pao subclasses. We obtained comparable results analyzing with the same software the *P. papatasi* and *L. longipalpis* transcriptomes (Additional file 6: Table S4).

#### Prediction of putative long non-coding RNAs

The Annocript annotation pipeline performs also a prediction of the putative long non-coding RNAs (lncRNAs) through the Portrait software, which uses support vector machine and is optimized for non-model organisms [41]. In brief, a transcript is annotated as non-coding if it has (i) no match in public databases, (ii) a Portrait non-coding probability > 0.95, (iii) a transcript length > 200 bp and (iv) an ORF < 100 aa. Using Annocript, we predicted 1229 putative lncRNAs in *P. perniciosus*. However, to obtain a more reliable prediction of the lncRNAs, we combined the Portrait results with the predictions from two other CPAT [42] and CPC [43] which use logistic regression and machine-learning methods, respectively. We set a very stringent threshold in both cases (see Methods). When considering the intersection among the three prediction methods we predict as putative lncRNAs of *P. perniciosus* 981 PERNI transcripts (Fig. 2) with a length range of 201–1575 bp, a N50 value of 340 bp and a GC content of 35.04 %, lower than that of the whole PERNI data set (42.12 %), in agreement with the low GC content of the lncRNAs described in other insect species [36]. Low GC content is emerging as a feature of the long non-coding transcripts, at least in humans where they are mainly studied [44]. This finding supports the reliability of our prediction (Additional file 7: Table S5).

#### Prediction of putative novel protein coding transcripts

In addition to the 11,736 annotated PERNI transcripts and the 981 putative lncRNAs, the PERNI data set contains 244 unannotated PERNI transcripts (Fig. 2) with an open reading frame longer than 100 aa (mean ORF length = 392.3 +/− 213.5), a GC content of 42.97 % and predicted as coding by Portrait (coding probability >80 %), CPAT (coding probability >80 %) and CPC (Score > 0) software (Additional file 7: Table S5). A TBLASTX analysis against the *P. papatasi* transcriptome revealed that 86 out of 244 PERNI transcripts have a highly conserved match (*E*-value < e-20; identity range 32–96 %) (data not shown). These PERNI transcripts might encode for novel proteins specific of the subfamily Phlebotominae and not present to date in the public databases.

### Expression level analysis

The relative expression level of the assembled PERNI data set was assessed in pooled Illumina reads of males and females, by the FPKM metric (Fragments Per Kilobase of transcript per Million mapped reads) [45] using the RSEM software [46]. In the PERNI data set FPKM values vary from less than 1 to 73,797 (median = 1.45, mean  = 28.94, standard deviation = 475.7) suggesting a wide range of expression levels (Additional file 8: Table S6). We classified PERNI transcripts as: (i) not expressed if FPKM was <1 (41.44 % in males and 44.77 % in females), (ii) weakly expressed if 1 < FPKM < 10 (31.72 % in males and 28.83 % in females), (iii) moderately expressed if 10 < FPKM < 100 (23.24 % in males and 22.98 % in females), and highly expressed if FPKM > 100 (3.59 % in males and 3.43 % in females). Overall, we concluded that vast majority of transcripts are weakly or not expressed, while only a very small fraction is highly expressed, with no substantial differences between males and females (Pearson’s Chi-squared test *p*-value = 0.21). Among highly expressed transcripts (FPKM >100) we found a significant enrichment (*p* < 0.01) for 77 GO terms in females and 55 in males (Additional file 9: Table S7), suggesting that highly expressed transcripts regulate sex-specific functions.

To validate the results of the *in silico* expression analysis, we selected ten transcripts in the PERNI data set and evaluated their relative expression by Real Time PCR (qPCR). In particular, we selected four PERNI transcripts with similar FPKM values in both sexes, three PERNI transcripts, which seems to be male-biased and three apparently female-biased according with their FPKM values (Additional file 10: Table S8). We included the *P. perniciosus* orthologs of the *apyrase* (Corset cluster-6143.0) and the *Act-3* genes (Corset cluster-14748.2) which in mosquitoes are known to be female- and male-biased, respectively [47, 48]. The first crucial step of the relative qPCR technique is the selection of the best transcript to use as reporter in the subsequent normalization. For this reason, we selected five putative reporter transcripts (see Methods), analyzed their expression levels by qPCR and examined the results using the NormFinder software [49]. Among the five putative reporters selected, *sod* (encoding for superoxide dismutase) and *gpdh* (encoding for glycerol-3-phosphate dehydrogenase) were observed to be the most stable between adult males and females of *P. perniciosus*. Consequently, we decided to use both as reference genes in our relative quantization experiments. The normalized expression values (Rn) of the selected transcripts of *P. perniciosus* in males and females were compared to the respective normalized FPKM values (nFPKM), resulting in statistically significant positive correlation. In particular, the Pearson correlation coefficient *r* was 0.743 in males (*p* = 0.014) and 0.862 in females (*p* = 0.001), showing a good agreement between the results obtained from the *in silico* and *in vivo* expression analysis. This is the first report on the selection of reporter genes in adult males and females of *P. perniciosus* and this result will be useful in all future studies on gene expression in this species.

### Differential expression analysis

Sexual dimorphism is marked between male and female sand flies with many morphological, behavioral, and physiological traits, which are typical for each sex. In particular, females are usually heavier than males while males have prominent external terminalia, with a relatively minute and slight abdomen compared to the females. Furthermore, closely related sand fly species can often be properly identified only using the species-specific morphology of sex-specific traits such as the dilatation of the distal part of the spermathecal ducts in females or the morphology of the copulatory valves (aedeagus) in males [50, 51]. Complex sexually dimorphic phenotypes in animals are mainly the result of differential expression between males and females of the same gene [52], however nothing is known in *P. perniciosus*. Therefore, to begin the study of the molecular bases of the sexual dimorphism in sand flies we interrogated our data set to identify genes differently expressed between adult males and females of *P. perniciosus*.

**Fig. 4:**
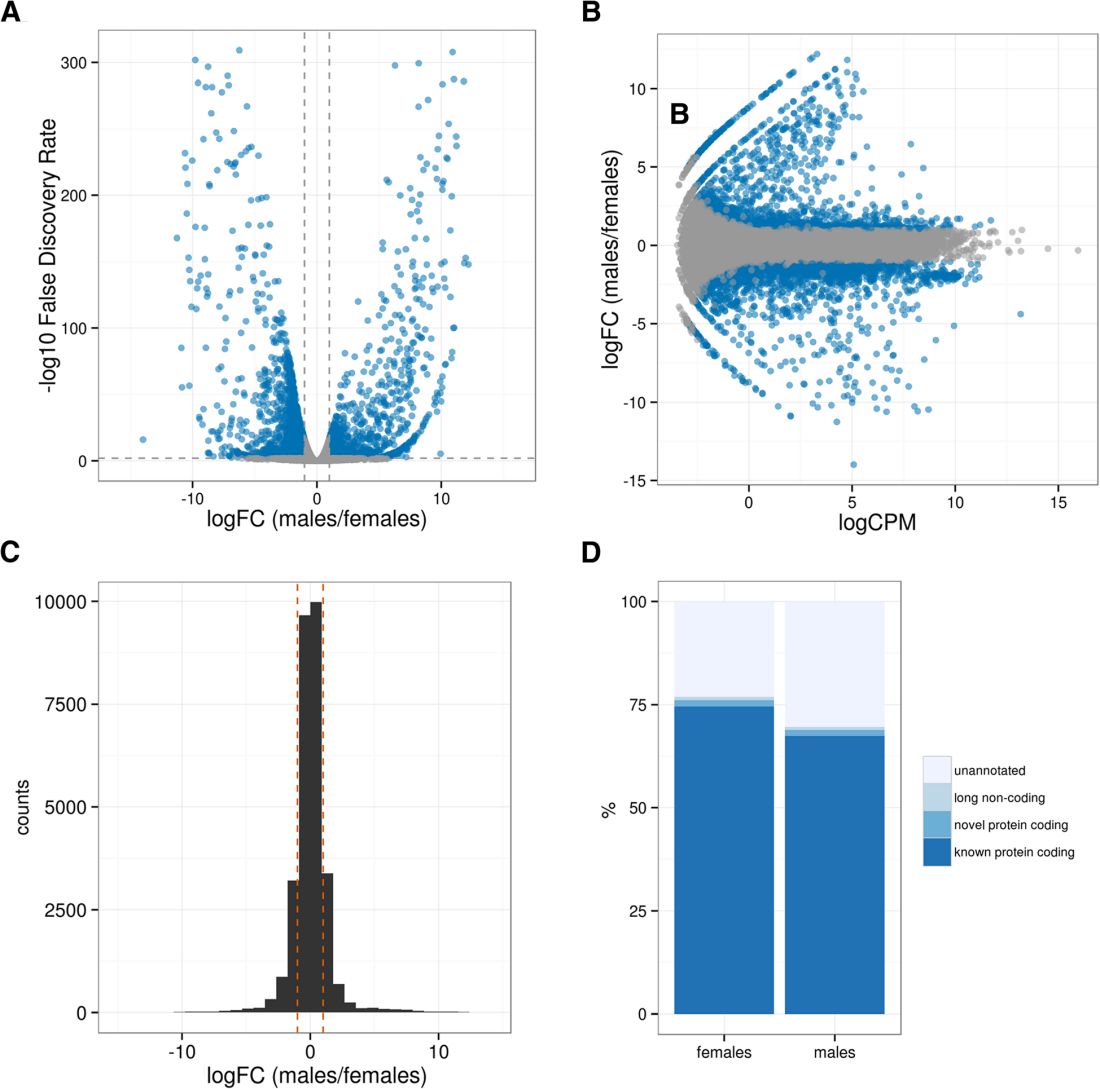
Differential expression analysis. Differential Gene Expression Analysis of adult male vs. female whole bodies of *P. perniciosus*. **a** Volcano plot showing the relative expression levels of the transcripts in adult male versus adult female whole body. The x-axis represents the log2 of the expression fold change (males vs. females) for each transcript of the PERNI data set. The y-axis represents the negative log10 of the adjusted *p*-value (FDR) of the Fisher’s Exact test. Blue data points (*n* = 8590) represent the transcripts that were statistically significant sex-biased (FDR < 0.01; dotted horizontal line). Dotted vertical lines represent the biological cut-off of 2-fold change in expression between males and females. **b** MA-plot showing the relative expression levels of the transcripts in adult male versus adult female whole body. The x-axis represents the log2 of the counts per million of mapped reads (CPM) for each transcript of the PERNI data set. The y-axis represents the log2 of the expression fold change (FC) for each transcript of the PERNI data set. Blue data points (*n* = 8590) represent the transcripts that were statistically significant sex-biased (FDR < 0.01; dotted horizontal line). **c** Histogram of the log2 of the expression fold change (FC) values between the 8590 sex-biased PERNI transcripts. Dotted orange vertical lines represent the biological cut-off of 2-fold change in expression between males and females. **d** Stack plot of the distribution of the annotation types of the sex-biased PERNI transcripts

We performed a differential gene expression (DGE) analysis between males and females by using the edgeR software [53] as implemented in the Trinity package. We used the gene-level counts for each biological replicate obtained by the Corset software. The quality control, performed with Trinity scripts (see Methods), revealed the high quality of the replicates, with low level of outliers and high correlation within each group (Additional file 11: Figure S3). The DGE analysis identified 8590 PERNI transcripts (29.3 % of the total transcripts; 4139 male-biased and 4451 female-biased) differentially expressed (DE) between sexes (FDR < 0.01) with a fold change (FC) ranging from 1.26 to more than 16,000 (Fig. 4a, b and c). The sex-biased Corset clusters, the corresponding PERNI transcripts, the mapped read counts, the FPKM values and their annotations are listed in the Additional file 12: Table S9. As a positive control, the *P. perniciosus* orthologs of the *apyrase* and *Act-3* genes, previously investigated, are present among the statistically significant DE genes in agreement with their sex-biased expression pattern in mosquitoes [47, 48].

The vast majority of sex-biased transcripts (75 %, 6111 out of the 8590; 2791 male-biased and 3320 female-biased) have a functional annotation in the UniprotKB database. This figure represented 52 % of the 11,736 annotated transcripts. In addition, 64 out of 8590 sex-biased transcripts were annotated as lncRNAs (29 male-biased and 35 female-biased) and 125 out of 8590 were annotated as putative novel protein coding genes (60 male-biased and 65 female-biased) (Fig. 4d). A RNA-seq study conducted on whole body adult samples of *Drosophila melanogaster* revealed that the percentage of the male-biased annotated transcripts is 20.7 % and the female-biased is 8.3 % [54]. This higher percentage of male-biased genes was attributed to the transcriptional complexity of the testes of the fruit fly [54]. In *P. perniciosus* the percentage of the annotated male-biased genes (21.3 %) is very similar to that observed in *Drosophila*. Conversely, in *P. perniciosus* we observed a much higher percentage of annotated female-biased genes (25.4 %) relative to male-biased genes. This result is in agreement with the sex-biased expression in sugar-fed adults of the mosquito *Anopheles gambiae*, where the percentage of the female-biased genes is higher than that of the male-biased [55].

To identify gene classes enriched in the sex-biased PERNI transcripts we selected the annotated male- and female-biased PERNI transcripts with a FC > 2 (1103 and 1449 PERNI transcripts, respectively) and we performed a GO term enrichment analysis for each sex using a R plugin of the Annocript software and applying the Fisher Exact Test (adjusted *p*-value < 0.01). We identified 80 enriched GO terms in females and 56 in males (Additional file 13: Tables S10). Among the enriched terms of females, most are involved in RNA metabolism, translation and oogenesis. In males, most of the enriched terms are related to transcription, signal transduction and response to stimuli (Additional file 14: Figure S4). Interestingly, among the male enriched GO terms we observed the monooxygenase activity (GO:0004497). Male-specific insect monooxygenases were found in the male reproductive system of *Blattella germanica* [56], *D. melanogaster* [57] and *Ips paraconfusus* [58] but their role remains unknown. Considering the importance of this enzyme family in the development of insecticide resistance in insects, it deserves further attention in future studies.

As a validation of the DGE analysis, we examined the sex-specific expression pattern of the top 31 DE PERNI transcripts (20 male- and 11 female-biased), chosen to have FDR value equal to zero and an FPKM value > 1 in at least one sex (Additional file 15: Table S11), performing a semi-quantitative RT-PCR experiment. Only 8 out of 31 of the selected DE PERNI transcripts have a functional annotation (six among female-biased PERNI transcripts, including *phenoloxidase*, *peroxidase* and a *vitellogenine receptor*, and two among the male-biased PERNI transcripts, *Trypsine* and *Glycerol kinase*).

**Fig. 5:**
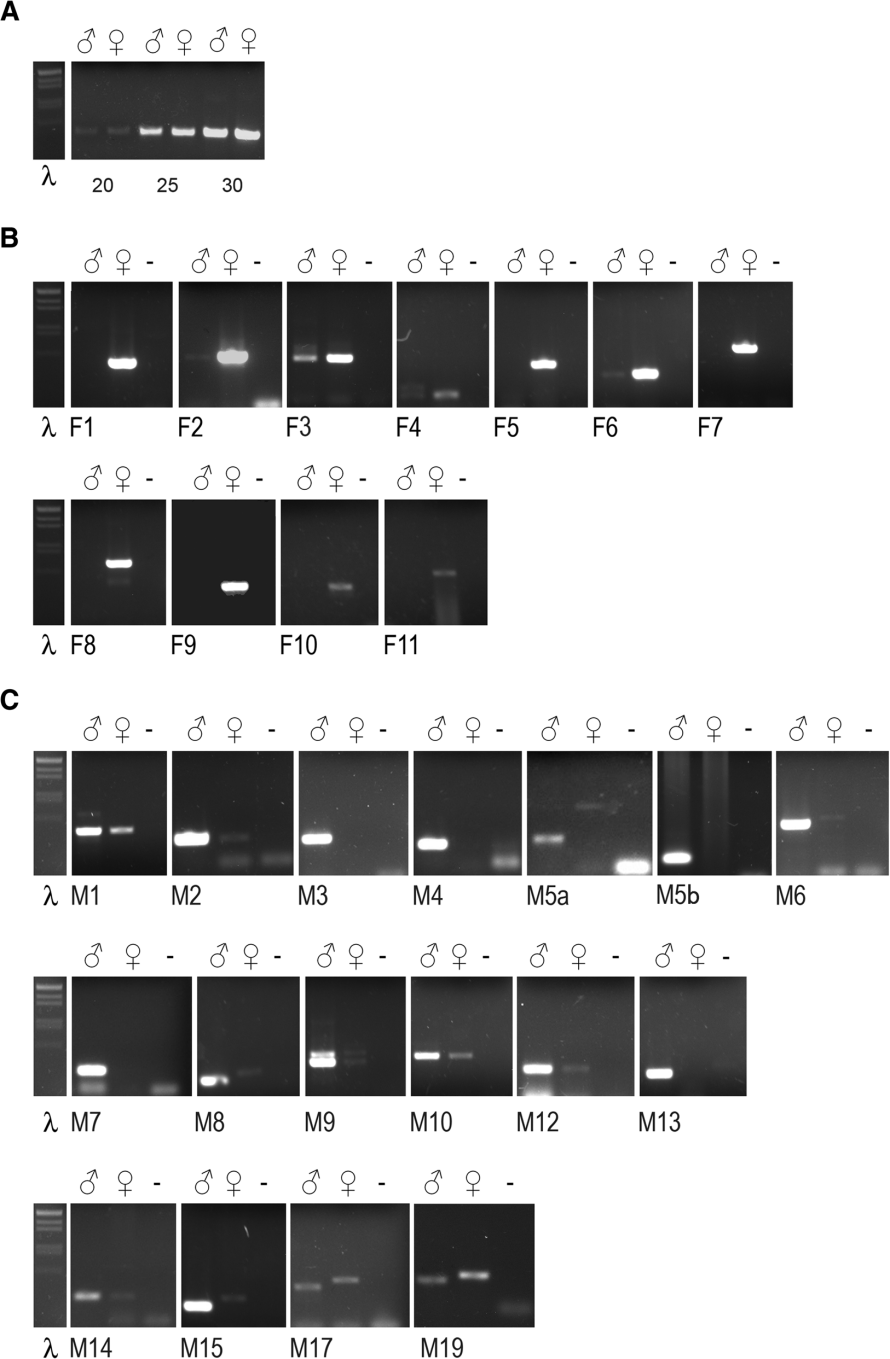
Semi-quantitative RT-PCR analysis of the top sex-biased PERNI transcripts. Semi-quantitative RT-PCR analysis of selected transcripts of male and female adults of *P. perniciosus*. **a** Normalization control using primer pairs for the *sod* gene of *P. perniciosus* at 20, 25 and 30 PCR amplification cycles. **b** Amplification of female-biased PERNI transcripts. For the transcript F4 we observed an extra male-specific amplification signal of larger molecular size. **c** Amplification of male-biased PERNI transcripts. For the transcript M1 we observed an extra male-specific amplification signal of larger molecular size and for the transcripts M5a, M6, M8, M15, M17 and M19 we observed extra female-specific amplification signals of larger molecular size. For the transcript M9 we observed an extra non-sex-specific amplification signals of larger molecular size. λ indicates the molecular weight marker (λ genomic DNA EcoRI-HindIII digested)

We obtained an amplification product of the expected molecular size for all the female-biased and 17 out of 20male-biased DE PERNI transcripts, confirming the quality of our DGE analysis (Fig. 5). The remaining three male-biased DE PERNI transcripts did not produce any amplification product. We hypothesize that this could be due to errors in the assembly of the three transcripts.

Interestingly, in some PCR amplifications we obtained unexpected additional fragments. In particular, we got a male-specific larger fragment in the DE PERNI transcripts F4 and M1; a female-specific larger fragment in the DE PERNI transcripts M5a, M6, M8, M15, M17 and M19; a non-sex-specific larger fragment in the DE PERNI transcript M9. We examined the unclustered Trinity assembly output to search for the presence of alternative isoforms of these nine DE PERNI transcripts and we found assembled alternative isoforms only for the M9 and M17 transcripts (data not shown). To verify the nature of the eight sex-specific alternative amplified fragments, we performed sequencing experiments, obtaining positive results for five DE PERNI transcripts (F4, M5a, M8, M15 and M17). The numerous attempts to clone and sequence the alternative fragments of M1, M6 and M19 DE PERNI transcripts have failed. The direct sequencing of the alternative fragments of the DE PERNI transcripts F4, M5a, M8, M15 and M17, revealed the presence in each of them of an additional nucleotide sequence with conserved 5’ and 3’ intron consensus sites (5’ = AG/GURAGU and 3’ = (Y)_n_NCAG/G) (Additional file 16: Figure S5). This result suggests that these additional amplification fragments might be produced by sex-specific alternative splicing events, via intron retention rather than alternative exon usage. Further analyses are required to clarify if the postulated sex-specific alternative splicing is somatic or germinal. These five transcripts have not been annotated by our analysis pipeline neither as coding nor as non-coding, but the presence of sex-specific alternative splicing let us hypothesize they might have a functional role.

### Evolutionary analysis

Non-synonymous and synonymous substitution rates and their ratio Ka/Ks can be used to infer the selective pressure acting on the nucleotide coding sequences. In particular, a Ka/Ks value lower than one indicates that the compared sequences are subjected to purifying constraint for amino acid substitutions. Conversely, when the Ka/Ks value is equal or higher than one the sequences are evolving neutrally or under positive selection, respectively.

To evaluate the evolutionary forces acting on the orthologous genes between two Phlebotominae species belonging to the same and to different genera, we measured the pairwise Ka/Ks ratio from the back-translated codon alignment of 3159 putative orthologs between *P. perniciosus* and *P. papatasi* (P-P) and of 3932 putative orthologs between *P. perniciosus* and *L. longipalpis* (P-L). Putative orthologs between species were identified by pairwise BLASTP analysis (*E*-value: 1e-6; min. hit coverage 70 %) and aligned using the software ParaAT [59]. For all genes Ka/Ks was always < 1 with mean Ka/Ks very similar for both comparisons (P-P: average Ka/Ks = 0.061, max = 0.794; P-L: average Ka/Ks = 0.064, max = 0.576, Additional file 17: Table S12) showing that a strong purifying selection is acting on the ortholog genes in both the comparisons.

**Fig. 6:**
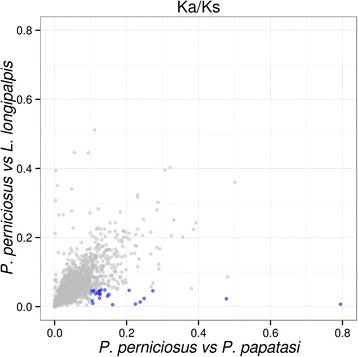
Ka/Ks plot. Plot representing the distribution of the Ka/Ks values between *P. perniciosus*/*L. longipalpis* and *P. perniciosus*/*P. papatasi*. Blue dots represent the ortholog pairs showing relaxation of selective constrains in the comparison *P. perniciosus*/*P. papatasi*

Merging of the two orthologous data sets yielded 1796 putative orthologs genes common to the three Phlebotominae species. For 25 of them Ka/Ks value is higher than 0.1 between *P. perniciosus* and *P. papatasi* and lower than 0.05 between *P. perniciosus* and *L. longipalpis* indicating a relaxation of the selecting constrains on these genes in the two *Phlebotomus* species when compared to *L. longipalpis* (Fig. 6). Interestingly, of these 25 genes, 17 are sex-biased in *P. perniciosus* (ten female- and seven male- biased, respectively). These findings are in agreement with the hypothesis that the relaxation of purifying selection, more than the positive selection, might be associated to sex-biased gene expression and drive phenotypic evolution, as described for *Solenopsis invicta* and *Apis mellifera* [60]. Further studies are required to confirm these preliminary results, focusing the evolutionary analyses on wild populations of *P. perniciosus* and/or extending the comparison to other phylogenetically related insect species.

## Conclusions

We presented the first adult reference transcriptome of *P. perniciosus* and its differential expression and evolutionary analysis. Our results represent a relevant resource for functional studies in the sand fly and provides baseline knowledge for future studies on sex-specific gene expression and pathways. Furthermore, our data will be useful to perform comparative analyses among phlebotomine sand flies and other insect species, e.g. mosquitoes, to dissect regulatory and biochemical pathways involved in arthropod blood feeding, host-parasite interactions, reproductive biology and somatic sexual differentiation. Finally, the annotated genes constitute an important toolkit that could enhance the discovery of new potential targets for vector-based control strategies against leishmaniases and other arthropod-borne diseases.

## Methods

### RNA extraction, quality control and sequencing

The adult individuals of *P. perniciosus* used in this study were from a laboratory colony held at Istituto Superiore di Sanità, Rome, Italy. This colony was founded with individuals originally collected in Spain. Sand flies have been reared in standard conditions at 28 °C, 80 % relative humidity and 12:12 h light–dark regimen [61]. Three pools for each sex (30 virgin males and 23 virgin sugar-fed emales, 7–10 days old) were utilized to extract total RNA with PureLink® RNA Mini kit (Life Technologies, Carlsbad, CA, US) according to manufacturer’s instructions. The integrity and purity of extracted total RNA were assessed using the NanoDrop 2000c (Thermo Fisher Scientific, Waltham, MA, US) and the Agilent 2100 Bioanalyzer with RNA 6000 Nano kit (Agilent, Santa Clara, CA, US). All the RNA samples had a A260/280 ratio of 2.2 and a RIN value higher than 7. About 1.5 micrograms of total RNA for each sample were sent to EMBL Genomics Core Facility in Heidelberg, Germany, for library construction and Illumina sequencing. Six strand-specific RNA sequencing libraries were produced using TruSeq Stranded mRNA Library Prep Kit (Illumina Inc., San Diego, CA, US) and used for 50 bp paired-end (PE) sequencing on two lanes of an Illumina Hiseq 1500, multiplexing the six libraries on each lane. We obtained a total of 277,105,235 PE reads. The raw data files were deposited into the Short Read Archive (SRA) of the NCBI (Accession Number: SRP059770).

### *De novo* transcriptome assembly and clustering

The stranded PE reads from the six libraries were pooled together and *de novo* assembled using Trinity (release 2014-07-17) [30, 31] with default parameters on the ADA Server at the Department of Biology, University of Naples Federico II (24 cores, 256 GB of memory). We applied the “trimmo” filtering and assembling pipeline as described in Salvemini et al., 2014 [36] without the mitochondrial and ribosomal transcripts depletion steps. Raw read pairs from each Illumina library were mapped back individually to the Trinity assembled transcriptome using the Bowtie software [62] allowing for multiple reads mapping (−a parameter) and the alignment outputs were stored as six separate BAM files. The clustering of the assembled transcripts was performed using the Corset software v1.02 [32] with default parameters. The Corset algorithm, using the read sets that have been multi-mapped to the *de novo* assembled transcriptome, hierarchically clusters the transcripts based on the proportion of shared reads and of transcripts expression patterns. This allows for discrimination between genes that share sequence, such as paralogs, if the expression levels between the compared groups are different. Then, the Corset software outputs gene-level counts, that can be easily tested for differential expression using count-based frameworks such as edgeR [53] and DESeq [63], and it consistently performs well compared to alternative clustering methods on a range of metrics [32].

### Functional annotation

To investigate and summarize the functional categories present in the PERNI data set, we applied the Annocript pipeline (https://github.com/frankMusacchia/Annocript) [38] using the UniProtKB reference database (2014–11 version) and the longest transcript of each Corset cluster as representative (Corset transcripts, PERNI data set). The Annocript pipeline performs various similarity searches using a speed-optimized BLASTX and BLASTP parameters and a custom parallelization of RPSBLAST to achieve a faster execution. It produces a final readable annotation table with assigned proteins, domains, GO terms, Enzymes, pathways, short and ribosomal RNAs, longest ORF size and non-coding potential. In particular, we performed the following similarity searches: BLASTX against TrEMBL/UniRef and SwissProt (parameters: evalue 1E-5, threshold 18, wordsize 4), RPSBLAST against CDD profiles (parameters: evalue 1E-5), BLASTN against Rfam and rRNAs (parameters: evalue 1E-5). To perform the comparative analysis of the GO terms distribution of the PERNI data set with the GO terms of the two sand fly species *L. longipalpis* and *P. papatasi* we downloaded the transcript data set from VectorBase (Lutzomyia-longipalpis-Jacobina_TRANSCRIPTS_LlonJ1.1 and Phlebotomus-papatasi-Israel_TRANSCRIPTS_PpapI1.1, respectively); the two data set were re-annotated using the Annocript pipeline with the same parameters as for the PERNI data set.

### Abundance estimation and differential expression analysis

We applied the RSEM software [46] and the Bowtie aligner [62], as implemented in the Trinity software package, to assign reads to the Corset clusters and to compute expression levels using the FPKM (Fragments Per Kilobase of transcript per Million fragments mapped) metric. We utilized the matrix file containing the mapped read counts for each of the three biological replicates for each sex, produced by the Corset software, to perform a differential gene expression analysis between adult males and females of *P. pernicious.* Replicate quality control was performed using the PtR Trinity perl script (release 2014-07-17). We applied the edgeR software [53] which uses a negative binomial model for differential expression analysis. We performed the analysis using the Corset clusters and a significance false discovery rate (FDR) threshold value < 0.01.

### Validation of RNA-seq results by qRT-PCR

Total RNA was separately extracted from pooled males and females of *P. perniciosus* (50 and 25 individuals, respectively), different from those used for the RNA-seq analysis, using the extraction and quantification protocol described above. Total RNA was quantified using Qubit fluorometer (Life Technologies, Carlsbad, CA, US) and reverse transcription reaction was conducted on 200 ng of male or female total RNA using the EuroScript Reverse Transcriptase kit (Euroclone, Pero, IT) with random examers, in a final volume of 20 μl. Five transcripts encoding for *superoxide dismutase* (*sod*) (Cluster-5663.0), *glycerol-3-phosphate dehydrogenase* (*gpdh*) (Cluster-15017.0), *cytochrome P450* (*cytp450*) (Cluster-16737.0), *glyceraldehyde-3-phosphate dehydrogenase* (*gapdh*) (Cluster-19016.0) and the gene for the hypothetical protein P119 (*p119*) (Cluster-13349.0) similar to the NOT3 protein of *Drosophila* were selected to evaluate the best reporter gene to use in relative quantization Real Time PCR experiments. Primer pairs utilized are listed in Additional file 18: Methods S1. Real Time PCR amplifications were conducted on 1/40 v/v of male or female reverse transcription reaction using the Brilliant III Ultra-Fast SYBR Green QPCR Master Mix (Agilent, Santa Clara, CA, US). The reactions were performed in technical triplicates. PCR efficiency (E) and threshold cycle (C_T_) for each well was calculated using the online tool RealTime PCR Miner [64] and the NormFinder software [49] was used to evaluate the best reporter gene. Real Time PCR reactions were conducted as described above. The mean relative expression ratio (Rn) and standard deviation of the ten selected transcripts in males and females were calculated using *sod* and *gpdh* as endogenous control genes by applying the formula Rn = (1 + E_target gene_)^-CT target gene^/(1 + E_control genes_)^-CT control genes^, where the E and C_T_ values of the control genes are the geometric mean of the efficiency and threshold cycle between *sod* and *gpdh*. The FPKM counts of the selected transcripts were normalized relative to the FPKMs of the transcripts *sod* and *gpdh* (nFPKM). The Pearson correlation coefficient *r* between the Rn and nFPKM values of males and females was separately calculated using the R package (www.r-project.org/).

### Semi-quantitative RT-PCR validation of sex-biased genes

Total RNA was extracted from pools of 50 adult males and 25 adult females using the extraction and quantification protocols previously described. Oligo-dT-primed cDNAs were prepared starting from 1 μg of total RNA using the EuroScript M-MLV Reverse Transcriptase (Euroclone, Pero, IT) following the manufacturer’s instructions. 1/40 v/v of the synthesised cDNAs were amplified by PCR in semi-quantitative conditions as described in Salvemini et al., 2006 [65] using the *sod* gene of *P. perniciosus* as reference. RT-PCR products were analysed by 1 % agarose gel electrophoresis. PCR products were gel-purified and sequenced with the Applied Biosystem BigDye 1.1 sequencing kit. Primers utilized in RT-PCR validation of the selected sex-biased transcripts are listed in the Additional file 18: Methods S1.

### Evaluation of coding potential

The prediction of coding potential of transcripts not annotated in the PERNI data set was performed, as described in Salvemini et al., 2014 [36], using three independent prediction methods: the Portrait software (performed in this paper as a plugin within the Annocript pipeline) [38, 41], the Coding Potential Calculator (CPC) [43] and the Coding Potential Assessment Tool (CPAT) [42]. In order to identify in the PERNI data set the potential non-coding transcripts with a high reliability, we selected stringent thresholds for each prediction method. Portrait-based prediction (YES), CPC coding potential score < −1.0 and CPAT coding probability < 0.05. Only those transcripts in accordance with the three conservative cut-off values were considered as putative non-coding transcripts of *P. perniciosus*.

### Evolutionary analysis

The putative coding sequence (CDS) of each PERNI transcripts of *P. perniciosus* was predicted using TransDecoder implemented in Trinity, with the default parameters setting. In order to select among the CDSs predicted by TransDecoder those encoding for the amino acid sequences annotated by Annocript, the reciprocal BLASTP best-hits between the two amino acid data sets were obtained using an *ad-hoc* perl script and used to extract the corresponding CDSs from the nucleotide data set. The same approach was applied to the transcriptome of *P. papatasi* [26] and *L. longipalpis* [25].

Putative ortholog CDSs between *P. perniciosus* and *P. papatasi* and between *P. perniciosus* and *L. longipalpis* were identified performing a best reciprocal BLASTP search, retaining only the sequences aligned with a continuous region covering at least 70 % of the query sequence. The pair-wise nucleotide alignment of the putative orthologs based on their amino acid alignment was performed using the ParaAT software [59]. ParAT uses the software Epal2Nal to back-translate the aminoacid alignment in a codon aligment which can be used for further nucleotides substitution analysis. The non-synonymous (Ka) and synonymous (Ks) substitution rates for each ortholog pair between *P. perniciosus* and *P. papatasi* and between *P. perniciosus* and *L. longipalpis* were calculated using the YN approximate method [66] implemented in the KaKs_Calculator software [67].

### Availability of data and materials

The data sets supporting the results of this article are available in the National Center for Biotechnology Information (NCBI) repository (Sequence Read Archives: SRP059770).

## Additional files

Additional file 1: Table S1. Ortholog hit ratio calculation of the transcriptome of *P. perniciosus* respect to *D. melanogaster* and *P. papatasi*. (XLS 876 kb) Additional file 2: Figure S1. Amino acid multiple alignments of the salivary gland low abundance proteins. Clustal W multiple alignments of the (**A**) hyaluronidase, (**B**) pyrophosphatase and (**C**) adenosine deaminase proteins of Psychodidae and Culicidae species. (PDF 97 kb) Additional file 3: Figure S2. Annotation type distribution of the PERNI data set. Venn diagram distribution of the annotated PERNI transcripts among (**A**) the ENZYME, PFAM DOMAIN, GO and PATHWAYS categories and (**B**) the GO categories BP (Biological Processes), MF (Molecular Function) and CC (Cellular Component). (TIFF 399 kb) Additional file 4: Table S2. GO terms of the annotated transcripts of the PERNI data set. (XLS 594 kb) Additional file 5: Table S3. GO term enrichment analysis of the transcriptomes of *P. perniciosus*, *P. papatasi* and *L. longipalpis*. (XLS 59 kb) Additional file 6: Table S4. Summary of the RepeatMasker search for TEs in the sand flies transcriptomes. (XLS 42 kb) Additional file 7: Table S5. Putative long non-coding and novel protein coding genes of the PERNI data set. (XLS 340 kb) Additional file 8: Table S6. FPKM values of the PERNI data set. (XLS 2310 kb) Additional file 9: Table S7. GO term enrichment analysis of the transcripts of the PERNI data set with FPKM values > 100. (XLS 44 kb) Additional file 10: Table S8. Selected PERNI transcripts for qPCR validation of the *in silico* expression levels. (XLS 26 kb) Additional file 11: Figure S3. Correlation between sequencing replicates. The correlation analysis between the male and female read replicates was performed using the normalized FPKM values. (**A**) Principal component analysis. (**B**) Scattered plot of log2 FPKM values between replicates. (**C**) Pearson correlation values between replicates. (TIFF 480 kb) Additional file 12: Table S9. Annotation table of the sex-biased PERNI transcripts. (XLS 8646 kb) Additional file 13: Tables S10. GO term enrichment analysis of male- and female-biased PERNI transcripts with fold-change > 2. (XLS 47 kb) Additional file 14: Figure S4. Enriched GO term frequencies in males and females. Top GO enriched terms in female- and male-biased PERNI transcripts with FDR < 0.01 and FC > 2. BP (Biological Processes), MF (Molecular Function) and CC (Cellular Component). (TIFF 1289 kb) Additional file 15: Table S11. Selected PERNI transcripts for semi-quantitative PCR validation of the sex-biased expression. (XLS 51 kb) Additional file 16: Figure S5. Sex-specific alternative splicing isoforms of the validated sex-biased transcripts. Clustal-W alignments of the sex-specific isoforms of the validated transcripts are reported. The 5’ and 3’ sequenced consensus sequences are represented in underlined case. Nucleotides matching with the sequenced consensus sequences are in bold case. (PDF 90 kb) Additional file 17: Table S12. Pair-wise Ka/Ks calculation between *P. perniciosus* and *P. papatasi* and *P. perniciosus* and *L. longipalpis* set of orthologs. (XLS 2344 kb) Additional file 18: Methods S1. List of primers utilized in the present paper. (PDF 120 kb)
