# Supplementary material for: De novo assembly and sex-specific transcriptome profiling in the sand fly Phlebotomus perniciosus (Diptera, Phlebotominae), a major Old World vector of Leishmania infantum
Source: BMC Genomics. 2015 Oct 23;16:847. doi: 10.1186/s12864-015-2088-x (PMC4619268; doi:10.1186/s12864-015-2088-x)
Supplement: Additional file 2: Figure S1. — Amino acid multiple alignments of the salivary gland low abundance proteins. Clustal W multiple alignments of the (A) hyaluronidase, (B) pyrophosphatase and (C) adenosine deaminase proteins of Psychodidae and Culicidae species. (PDF 97 kb) [file 12864_2015_2088_MOESM2_ESM.pdf]

(A)

CLUSTAL W (1.99) multiple sequence alignment, Hyaluronidase protein

```
PpeHYALU_KT160228   MDFLCFLWFLVGILSFCTGSEILKSPERNFTIYWNVPTNQCNRHNYTSANETKPDFPTLL
PorHYALU_AGT96452.1 MDFQCFLWFLIGFLNFCIGSES-ENPEKNFTIYWNVPTDQCNRHNYT--NETKPNFPELL
PtoHYALU_AEK98519.1 MDFLCFLWFLFGFIGFCTGSETLKSPERNFTIYWNVPTDQCNRHNYTA-NETKPNFPELL
LloHYALU_AAD32195.1 MNWIFHLFCAV-YGIFCE--------ENSFTIYWNVPTHQCEKLNVS--------FISLL

PpeHYALU_KT160228   TNLSIVHNVNGSFRGEEFRILYSPGLWPSMEHNKTENGTHGGMPHHGDLEKHLEQLETDI
PorHYALU_AGT96452.1 TNLSIVHNVNGSFRGEEFRILYSPGLWPSMEHNKTENGTHGGMPHHGNLTEHLEQLEKDI
PtoHYALU_AEK98519.1 TNLSIVHNFNGSFRGEEFRILYSPGLWPSMEHNKTENGTHGGMPHHGNLTKHLEQLEKDI
LloHYALU_AAD32195.1 KELNIVHNKDGNFSGESFTILYSPGLWPSMEHKNITN---GGMPQCGNMTLHLEKLEKDV

PpeHYALU_KT160228   KNCSHINYIPEHFTGMAVIDMESWRPVFRQNTGWMQIYRKLVFEEIDRKACQNETYFKEI
PorHYALU_AGT96452.1 NNCSHINYIPNNFTGMAVIDMESWRPVFRQNTGWMEIYRKLVFREIDSNATLRNLVYNDS
PtoHYALU_AEK98519.1 KNCSNINYIPENFTGMAVIDMESWRPVFRQNTG---------------------------
LloHYALU_AAD32195.1 K----EKLKDDGYSGLAVIDMESWRPVFRQNTGWMIKYRELTFEQYN------KTLAEEY

PpeHYALU_KT160228   LNNTTCSGNKANVCFKEAAKIFEPMAIDYMNKSIAKVRELRPEAHWGYYGFPYCFNI--R
PorHYALU_AGT96452.1 SG-ITCEKNRTNLCFKEAAKIFEPMAIDYMNKSIAKLRELRPEAHWGYYGFPYCFNI--R
PtoHYALU_AEK98519.1 ------------------------------------------------------------
LloHYALU_AAD32195.1 KNNTTNDKLR-NRLIKESAEIFEAPAKDFLMNSTELVKKYWKDAKWGYYGFPYCFNMGVA

PpeHYALU_KT160228   KNDRNESCPKPVPKENNNTAWLFQSYDSWYPSVYISHDNFTEEDRQNLVSGRVKEYNRLR
PorHYALU_AGT96452.1 KDNRSESCAELVQKENDNTTWLFQSYNSWYPSVYISHDNFTEEDRLKLVRGRVQEYNRLR
PtoHYALU_AEK98519.1 ------------------------------------------------------------
LloHYALU_AAD32195.1 ANARNESCPKIVKEENNKTEWLFKSYDYWFPSVYITKVNFTCEERGQLVRGRVTEYQRLR

PpeHYALU_KT160228   NLTNQNATIYPYVWLLYNLDNRTEVYLNETDLNMTLTTLKNYTMDGAVIWGMSQNVNTSD
PorHYALU_AGT96452.1 NLTNQNATIYPYIWLLYNLDNRTEVYLNKTDLNMTLTTLKIT------------------
PtoHYALU_AEK98519.1 ------------------------------------------------------------
LloHYALU_AAD32195.1 KEFNPKAKIYPYVWFLYNLSN---EYLSKEDLEMSLKILKMGKMDGAVIWGSSKNLTKEC

PpeHYALU_KT160228   KCLKLYKYVNETLKPILEGLNITRHEPKSNGTGSILESQKCPKNTTNTTNNTNQRKKREI
PorHYALU_AGT96452.1 ------------------------------------------------------------
PtoHYALU_AEK98519.1 ------------------------------------------------------------
LloHYALU_AAD32195.1 ECKDLYDYVNGTMRTVLEGLKKPENNVKWNG--SCQETSDAARYLKNCTDKQ--------

PpeHYALU_KT160228   KEDAQCQNCLINDEPASPASNSDHSNQDSSIFFVKYLFQVSYNFFQSVFSQI
PorHYALU_AGT96452.1 ----------------------------------------------------
PtoHYALU_AEK98519.1 ----------------------------------------------------
LloHYALU_AAD32195.1 ----------------------------------------------------
```

(B)

CLUSTAL W (1.99) multiple sequence alignment, Pyrophosphatase protein

```
PpePYRO_KT160227       MFFEQGAKKPIGAAEKGKRDAKSQYFAAFSHVKMVNRS----EIR---------LFLSAL
ParPYRO_ABA12155.1     ---------------------------------MLNFT----GVS---------IAIVLW
PorPYRO_AGT96453.1     ---------------------------------MVNLS----EIR---------LFLSAL
PduPYRO_ABI20154.1     ---------------------------------MLLLV----E-----------AAVILW
AaePYRO_XP_001660105.1 ---------------------------------MVPSAIAPIEWPHRWLIFGGLLVMILG
AgaPYRO_XP_001688168.2 ---------------------------------MTTTVVVL-EVVQLVLLLLLPGKASSG
CquPYRO_XP_001848713.1 ---------------------------------MIPRSTARSRW-LV-VVAAVTLLHLLD

PpePYRO_KT160227       IVLLFATLRIVDGSQGNSLLVISYDAFRNDYLKRNITPYLNNFREEGVSVPFMKNVFPTK
ParPYRO_ABA12155.1     CAVTGSAARNV-GRQENSLLVISYDAFRNEYLQRNITSYMNSFQQQGVSAPYMKNVFPTK
PorPYRO_AGT96453.1     LVILFATLRIIDGSQGNSLLVISYDAFRNDYLKRNITPFLNNLREEGVSVPFMKNVFPTK
PduPYRO_ABI20154.1     CCII----KCITGCQENSLIVISFDGFRNDYLERNITPYLNAFREEGVSVPYVKNVFPSK
AaePYRO_XP_001660105.1 ASGVLASPSMTTSSAPAALIVVSYDAFRTEYLRRNSTAFMNELRRNGTTAEYLRNVFPTK
AgaPYRO_XP_001688168.2 GTVAGSAAASGSVQSPPVLIVVSYDGFRTEYLQRNSSAYINELRRNGTTSTHLRNVFPTK
CquPYRO_XP_001848713.1 GGAWCKRVRGNDPADETVLIVVSYDAFRTEYLRRGSASYMAELGHNGTWAEYLRNVFPTK

PpePYRO_KT160227       TFPNHHTIATGVFPGVHGVTANSVFDRVSGQKLEYGYPLFHYNEAIVPIWTLNEMRGHYS
ParPYRO_ABA12155.1     TFPNHHSIATGVFPGVHGVTANSVYDRVSGKYLNYGFPLFHHNEQVVPIYTLNELRGKYS
PorPYRO_AGT96453.1     TFPNHHTIATGVFPGVHGVTANSVFDRVSGQKLEYGYPLFHYNEAIVPIWTLNEMRGQYS
PduPYRO_ABI20154.1     TFPNHHTIATGVFPEVHGIMANALFDHKTEKLVYGYPLYHQNEAIIPIWTLNEMQGKHS
AaePYRO_XP_001660105.1 TFPNHHSIATGVYPNQHGVIANEIYDHLRG-KLEYSYEMYHFSDDIVPIWTLNELKGGHS
AgaPYRO_XP_001688168.2 TFPNHHSIATGVYPNVHGVMAGALYDAVQGP-LNYSYELFHFNPELLPIWALNQYAGGYS
CquPYRO_XP_001848713.1 TFPNHHSIATGVFPNQHGVMAGALYDHGRG-KINYSYDLFHFNEELVPIWTLNEQHGGHS

PpePYRO_KT160227       GCMMWPGSDFSYSGR--NCTYVVSYNKSIAWEDRVDTVMQWLSDPKKPANLVMMYFEDPD
ParPYRO_ABA12155.1     GCMMWPGSDFPYSGR--NCTHVQVYNKSMAWSERVDIVMNWLTDKEKPASLVMMYIEEPD
PorPYRO_AGT96453.1     GCMMWPGTDFPYSGR--NCTYVVSYNKSIAWEERVDTVMQWLSDPKKPASLVMMYFEDPD
PduPYRO_ABI20154.1     GCMMWPGSNFFYSGK--NCTYNIPYNKSIPWDERVDIALSWMTDKEKPANLLIMYFEDPD
AaePYRO_XP_001660105.1 GCMMWPGSNMPYTKAKVNCTYVKAYNMSLPWNDRVDTAFQWIRDPKQPANLVMLYIEEPD
AgaPYRO_XP_001688168.2 GCMMWPGSDFGYGSRNLTCSHTQPFNLSIPWTDRLDTVFRWIRDADRPANLIMLYIEEPD
CquPYRO_XP_001848713.1 GCMMWPGSDFPYTRGNITCSHLQPFNLTLPWNDRVDTAFRWIRDPTRPANLVMLYIEEPD

PpePYRO_KT160227       THGHIYGPDSNVIRDLIIKLDNLTRSIQQKLKEKNLTDRVNVIHLSDHGMEGVSSAKFID
ParPYRO_ABA12155.1     TDGHIYGPDSDVIRALIIKLDNLTKIIQEKLKENSLSDRVNVIHLSDHGMEGVTSKNVID
PorPYRO_AGT96453.1     THGHIYGPDSNVIRDLIIKLDNLTKSIQQKLKEKNLTDRVNVIHLSDHGMEGVSSAKFID
PduPYRO_ABI20154.1     SHGHTYGPNSDKVNEAITKVDNLVYSIQEKLKKKQLFDHVNIVFLSDHGMEGVIQDNFID
AaePYRO_XP_001660105.1 YYGHIYSPDSDRVAQLLIKLNDLTRYIYDKVREFNLQDRVNVVHLSDHGMDSLMPKNFIN
AgaPYRO_XP_001688168.2 YYGHIYGPESDRIEQLVVKLNDFTRQLHARIAEQRLAERVNVVHLSDHGMESVMPKNFIN
CquPYRO_XP_001848713.1 YYGHIYSPESDRVAQLVVKLNDLTRYIHDKIREFGLVERVNVLHLSDHGMDSLMPKNFIN

PpePYRO_KT160227       LRKAVTNGS-CEFYGTSPVLQIVPKDGK-FTEVYQNLTREAKTNGHFKVYSNDELLARWH
ParPYRO_ABA12155.1     LRKFVNNGT-CDFYGTSPALQIVPKPGQ-FDDVYQRLRRGAESNGHFKVYNDTELLERWH
PorPYRO_AGT96453.1     LHKAVTNGS-CEFYGTSPVLQIVPKDGK-FNEVYQNLTRDAKTYGHFKVYSNDELLARWH
PduPYRO_ABI20154.1     LYNFVAGAS-CDFYGSSPILQVVPKPGK-YDEVLCGLKKGAETNRHYKVFTKKKNPPLWH
AaePYRO_XP_001660105.1 LTSFVPTDVKYDRYGNTPVLQIVPKVKQQTADLYRALKNASEKNGNFDVYTLENLPARWH
AgaPYRO_XP_001688168.2 LTSLVSAELRYDTYGSTPVLQIVPKVEQDRSELYRQLRRAADADGRFEVYMVENLPARWQ
CquPYRO_XP_001848713.1 LTSFVPADMKFDVYGSTPVLQVVPKVKQQTADLYRALKNASEKSGNFDVYTLENLPTRWH

PpePYRO_KT160227       FNNSARSGPITALADPGYAFQDMYEAADWYAEKYNVSFTPQHEYGIHGYDNEGLPSMYSM
ParPYRO_ABA12155.1     FKNPARSGPITAVADPVYAFQDLYDSAAWYTANYNVTFTPDHVYGIHGYDNQGLPSMYSM
PorPYRO_AGT96453.1.    FNNSA-------------------------------------------------------
PduPYRO_ABI20154.1     YNNTERTGPITVLADPPYAFQDMFTAAENYKKTYNVSG-VNHTYGVHGYDNQAAPSMYSM
AaePYRO_XP_001660105.1 FNNSQRTGPITAVARLGYGFDDMWDTVEYYRKTYNVSVTPESKYGVHGYDN-DLQVMHPI
AgaPYRO_XP_001688168.2 YNNTRRTGPITAVAQHGYGFDDMWRTVEYYRKHFGVEVTPDTQYGMHGYDN-AQLAMNSI
CquPYRO_XP_001848713.1 FNNSQRTGPITAVAKLGYGFDDMWDTVEYYRKAFNVSVTSETKYGVHGYDN-ELPIMHPI

PpePYRO_KT160227       FMAKGPDFQQHKVLTPFDTVDLFSLFVKILNISNPPTTNGTLSHVTDALKSTIPSGSSR-
ParPYRO_ABA12155.1     FMAKGPDFQEHKVLEPFDSVDLYDLFVKILNISNPPVTNGTLAHVAAELKTSSLSNSTR-
PorPYRO_AGT96453.1     ------------------------------------------------------------
PduPYRO_ABI20154.1     FMAKGPDFRNRKVLLPFNTVDYYNLFAQLLNISNPPPTNGTLAHLKDALKTSDSSGIYR-
AaePYRO_XP_001660105.1 FFGYGPRIRERTMVDPFDTVDLYYLFCEILNLKPPSYLAGQRQHIAGVLRNDSRDDDGN-
AgaPYRO_XP_001688168.2 FFSYGPAIREQLTVEPFDTVDLYYLFCEILNLAPPNYLAGNVNHIQHILRNGSRDDDDDN
CquPYRO_XP_001848713.1 FFGYGPRIRERTTVEPFDTVDLYYLFCELLGLDAPYYLDGKREHIVAVLRNDSRDDDDD-

PpePYRO_KT160227       -----------KDPKVSAAIVARS------------------------------------
ParPYRO_ABA12155.1     -----------KNPKVSAAIVARS------------------------------------
PorPYRO_AGT96453.1     ------------------------------------------------------------
PduPYRO_ABI20154.1     -----------KY-----------------------------------------------
AaePYRO_XP_001660105.1 -----GDG--STRTGTLVVIFAGSLVGSFALVSMLAYVVLWQRRRRENMVPHYLYDEAES
AgaPYRO_XP_001688168.2 DPNGGGSSDATTRAETIAVIFAGSLIASFAIVSVFALGVIYQRRRREHMVPPYLYEETET
CquPYRO_XP_001848713.1 -----DPSGTSARTVTLMVIFAGSLLASFGLVSMLAYVVIWRRRRRENLVPHYLYDETES

PpePYRO_KT160227      -----------------------------------------------------
ParPYRO_ABA12155.1     -----------------------------------------------------
PorPYRO_AGT96453.1     -----------------------------------------------------
PduPYRO_ABI20154.1     -----------------------------------------------------
AaePYRO_XP_001660105.1 FLDEGNKLLPAAAHLQQQQSQYQHPPASNHRSHQHHHQLRSSASINGDVVSIDV
AgaPYRO_XP_001688168.2 FIDDG---------------NKMLHTSTTNSS------------INGDVVSVDV
CquPYRO_XP_001848713.1 FSGRS----------QQAAAGRRSPTATAAAT----------------------
```

(C)

CLUSTAL W (1.99) multiple sequence alignment, Adenosine deaminase protein

```
PpeADENO_KT160229        MLLKVVVWLLATSAVLAFDWTEFRMERK-YKDYLQKRSYYLAEEEDRSVGSDIELTAKEQ
LloADENO_AAF78901.1      MFSQLVVWLLATSTVCLAWDNSWIMDMK-YERYSQRRSYYLAEEEDRSVGSDIELTAKEQ
PduADENO_ABI20162.1      MFPRLIVWLLAASAVHAVLDISNIKPKRDYENFLQKYAEYADDEVDRSVGSDITLSLKEK
AaeADENO_XP_001651236.1  M-PRI-----------------------AYEDFLAKRAEFVNQEESRALGSDVVLNEDEQ
AgaADENO_XP_308848.4     M-ARP-----------------------AYDEFCRQREEFFAREQGRGLGANLVLSPSEQ
CquADENO_XP_001861718.1  M-SRL-----------------------TYEQFQAQRAEFLRDEESRALGADIVLTEDEQ

PpeADENO_KT160229        IVNERLMALKRKELAEGLENPAGFIPWNHMFDVLNRINSSEIFDILRRMPKGGILHAHDT
LloADENO_AAF78901.1      VVNERLMELKMTELKNGLQDPAGFIPWNHIFDVLYRINSSELFHIIQKMPKGGILHAHDT
PduADENO_ABI20162.1      FVNQYLMDLKTEELKAGLKNPSQFIPSNHFFSVLDRINSSEIFKIIRRMPKGAILHAHDT
AaeADENO_XP_001651236.1  KVNEYLMTLKKAELEEGFKSPITFAPSRHFFKVLDKIKNSPLFQLIQKMPKGGILHAHDT
AgaADENO_XP_308848.4     RLNQYVMHLKQQELAKGVENPYELVSARHFFEMLDRINESPLFRLIQKMPKGGVLHAHDT
CquADENO_XP_001861718.1  KVNEWLMKLKKTELDAGFKTPREFAPARHFFTVLEQIKASPLFQLIQRMPKGGILHAHDT

PpeADENO_KT160229        ALCSTDYVISLTYEPNLWQCTDPETGAL----SFKFSREAPTNTETCQWTSVAAERTKLG
LloADENO_AAF78901.1      ALCSTDYVISLTYEPNLWQCADPTTGAF----QFLFSREAPTNTDTCTWTLVADERAKQG
PduADENO_ABI20162.1      ALCSTDYVVSITYRDHLWQCADPKTGAL----QFRFSKESPKNTDTCQWTPVSEERKNQG
AaeADENO_XP_001651236.1  AIGSMETVIKATYQKNLWQNGEFGRASP---PHFKFSKTQPEPLDGVEWRSVSDVRKELG
AgaADENO_XP_308848.4     AIGSTELIVRATRHAHLWQSGSIPQTAADPMPVYTFSRAKPTA-EG-EWRLVADIRSTMG
CquADENO_XP_001861718.1  AIGSMETIVKATYREHLWQNGEFDRPTP---PNYKFSRTKPDPLDGVEWRSVADIRKELG

PpeADENO_KT160229        EENYNSGLRSQLSLYTTDPINHNRDVDSIWRQFMGIFGVNDGLLSYAPIWKAYYKQFLKE
LloADENO_AAF78901.1      EENYNSALRSQLSMYNTNPIMHNRDVDSIWRQFMGIFGVNGGLLTYAPVWKAYYLQFLKE
PduADENO_ABI20162.1      EEQYNSKLRSQLSLYNTDPINRSRDVDSIWNDFMGLFGVNFGLLTYAPVWKDYYKQFLKE
AaeADENO_XP_001651236.1  NEGFDQNLRELLSLFVPDPEVAYPCINHAWGRFFNMFISLEPIVTFKPVWEEYYTNTLKE
AgaADENO_XP_308848.4     DAAYEAAIRKMFTLYTTDPLNEHRDINDVWRKFMALFICFEPMVTYRPVWEEYYYGCLEE
CquADENO_XP_001861718.1  NEGFDQNLRDVFTLFDEEPSQAYSCINHIWGKFQYMFISLEPIVTYKPVWEDYFRNSLEE

PpeADENO_KT160229        MMEDGVQYLELRGTLPPLYDLDGKTYHEEEILHIYQDATREFKQENPTFIGAKFIYAPVR
LloADENO_AAF78901.1      MFADGVQYLELRTTLPPLYDLDGKTYNEVEIMQIYYDATKEFKKQNPTFIGAKIIYAPVR
PduADENO_ABI20162.1      MMEDGVQYLELRGTLPPLYDLDGKIYNEEQVVEIYYNVTEEFKKENSTFIGAKFIYAPVR
AaeADENO_XP_001651236.1  LYADNVTYLEFRGVLPPVYDLEGKIYSPEEVVQIYYDLSEKFKQEHPDFVGVKFIYAPIR
AgaADENO_XP_308848.4     LLADNVTYLEFRGLLPPVYDLDDRKYTPEEIVQMYVDQSEKFLRANPKFAGVKFIYAPLK
CquADENO_XP_001861718.1  VHQDNVCYLEFRGVLPAVYDLDNRVYTPEEVVQIYYDIVQNFKQTHPTFIGVKLIYAPIK

PpeADENO_KT160229        VVDDAGLPALMDKVRELHEQYPNFMAGFDLVGQEDKGRPLIDFSKEILGLPDSINFFFHA
LloADENO_AAF78901.1      VVDDAGIPALMAKVRELHEKFPDFMAGFDLVGQEDKGRPLIAFSREILKLPNSIDFYFHA
PduADENO_ABI20162.1      FVNATGIKTLTTTVKQLHERFPDFLAGFDLVGQEDKGGPLIGFSRELLELPESINFFFHS
AaeADENO_XP_001651236.1  AADDSTFDGYLQEAENLHKKFPTFVAGFDLVGQEDLGRPLTDFNERLLKMSPTIQFFFHA
AgaADENO_XP_308848.4     FCDDSTFDGYLTLVQTLKARFPTFIAGFDLVGQEDLGRPHTDFNERLLRLPAGINFFFHA
CquADENO_XP_001861718.1  FADDALFDTFLDTAESLHQKFPTFVAGFDLVGQEDTGRPMTDFNERLLRMSPTIQFFFHA

PpeADENO_KT160229        GETNWNGL-TDDNLIDAVLLGTKRIGHGYAVLKHPRVLKQVKRDKIALEVCPVSNQVLRL
LloADENO_AAF78901.1      GETNWDGM-TDDNLIDAVLLGTKRIGHGYAVLKHPRVLKEVKRNKIAIEVCPASNQVLRL
PduADENO_ABI20162.1      GETNWNGM-TDDNLIAAVTLGTKRIGHGYALFKHPRVLKQVKKDKIAIEVCPISNQVLRL
AaeADENO_XP_001651236.1  GETNWNGLSTDDNLIDAVLLGAKRIGHGFAAVKHPRVLEELKKRNICIELNPVSNQVLKL
AgaADENO_XP_308848.4     GETNWTGR-RDENLIDAILLGTKRIGHGFAAIKHPVVLEEIKKRNICIELNPISNQVLKL
CquADENO_XP_001861718.1  GETNWCGL-IDENLIDVILLGTKRIGHGFAAVKHPRVLEEIKKRNICIELNPISNQVLKL

PpeADENO_KT160229        VADMRNHPGAVLLANKEYPVVISSDDPSFWEAKPLTHDFYMAFLGLASERQDLRLLKQLA
LloADENO_AAF78901.1      VADYRNHPGSVLLANKEYPVVISSDDPSFWEAKPLSHDFYMAFLGLASSRQDLRLLKQLA
PduADENO_ABI20162.1      VADMRNHPGSILLANKKYPMVISSDDPSFWEATPLSHDFYMAFMGLASYHQDLRMLKQLA
AaeADENO_XP_001651236.1  VDDCRNHVGAIYFS-DSYPVVVSSDDPAFWCASPLSHDFYVAFLGLASARQDLRLLKKLA
AgaADENO_XP_308848.4     VQDFRNHPGGFYFS-DNYPVVVSSDDPSFWCASPLSHDFFVAFMGLASARADLRLLKQLA
CquADENO_XP_001861718.1  VDDYRNHVGAIYFS-DNYPVVVSSDDPAFWCASPLSHDFYMAFLGLAAARQDLRLLKKLA

PpeADENO_KT160229        INSIKYSAMTPLEKMNAMRLWEAEWKKFIEELSA-------
LloADENO_AAF78901.1      INSIKYSAMSPREKLQAMQMWEAEWKKFIDGFNA-------
PduADENO_ABI20162.1      INSLEYSSMTLEEKTNAMKLWEAEWEKFIKELETEVFSLLE
AaeADENO_XP_001651236.1  LNSIEYSGMSKAEKVEAKHKWSIAWNHFIDQTLKTQ-----
AgaADENO_XP_308848.4     LNSIEYSSMEDEEKTIATQKWTAAWNEYVEEMLKTIPAEF-
CquADENO_XP_001861718.1  LNSLEFSAMSKAEKAAEKLKWTVAWNSFIDQTVKSIA----
```
