# Supplementary figures and images for: De novo assembly and sex-specific transcriptome profiling in the sand fly Phlebotomus perniciosus (Diptera, Phlebotominae), a major Old World vector of Leishmania infantum
Source: BMC Genomics. 2015 Oct 23;16:847. doi: 10.1186/s12864-015-2088-x (PMC4619268; doi:10.1186/s12864-015-2088-x)

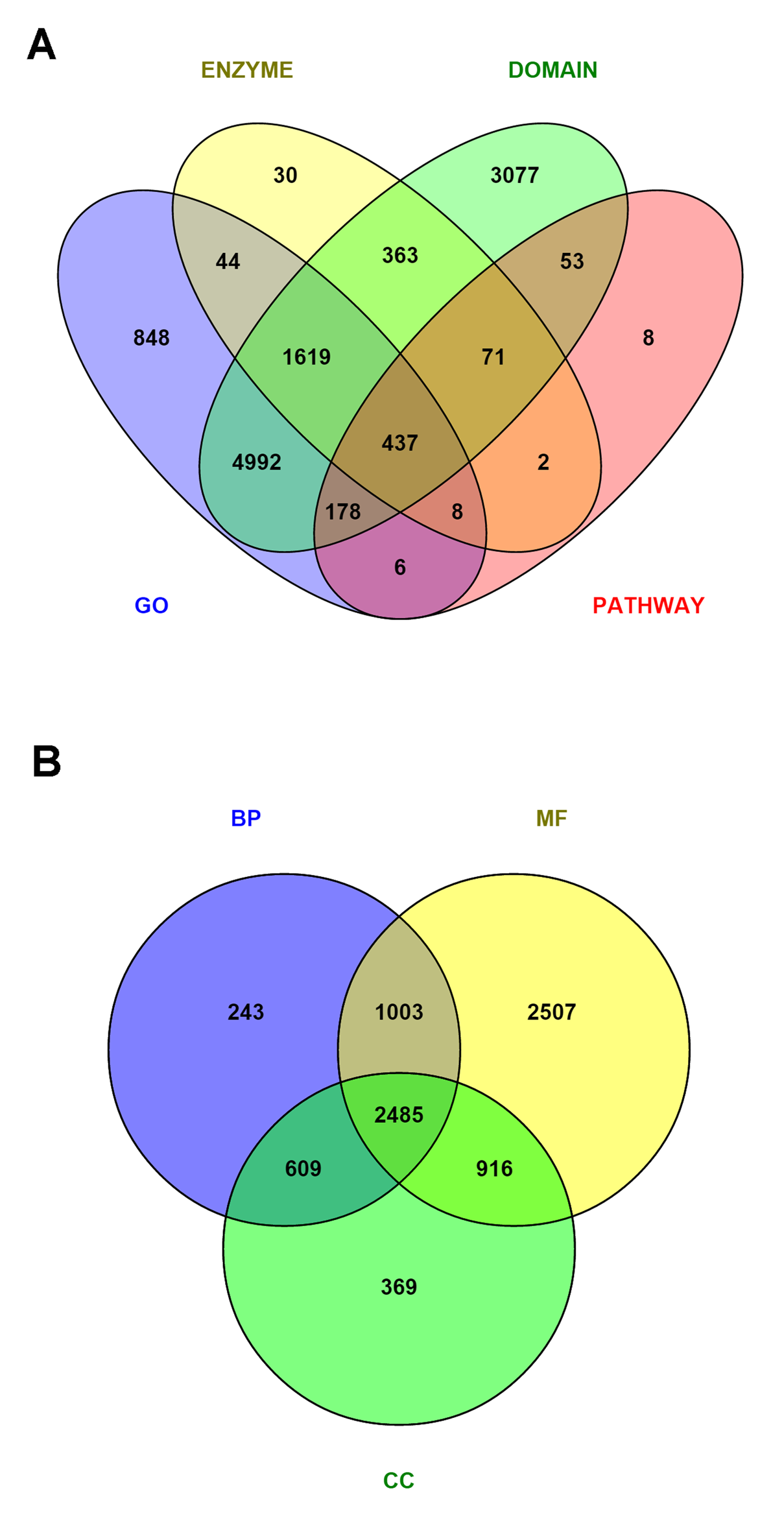

Supplement: Additional file 3: Figure S2. — Annotation type distribution of the PERNI data set. Venn diagram distribution of the annotated PERNI transcripts among (A) the ENZYME, PFAM DOMAIN, GO and PATHWAYS categories and (B) the GO categories BP (Biological Processes), MF (Molecular Function) and CC (Cellular Component). (TIFF 399 kb) [file 12864_2015_2088_MOESM3_ESM.tif]

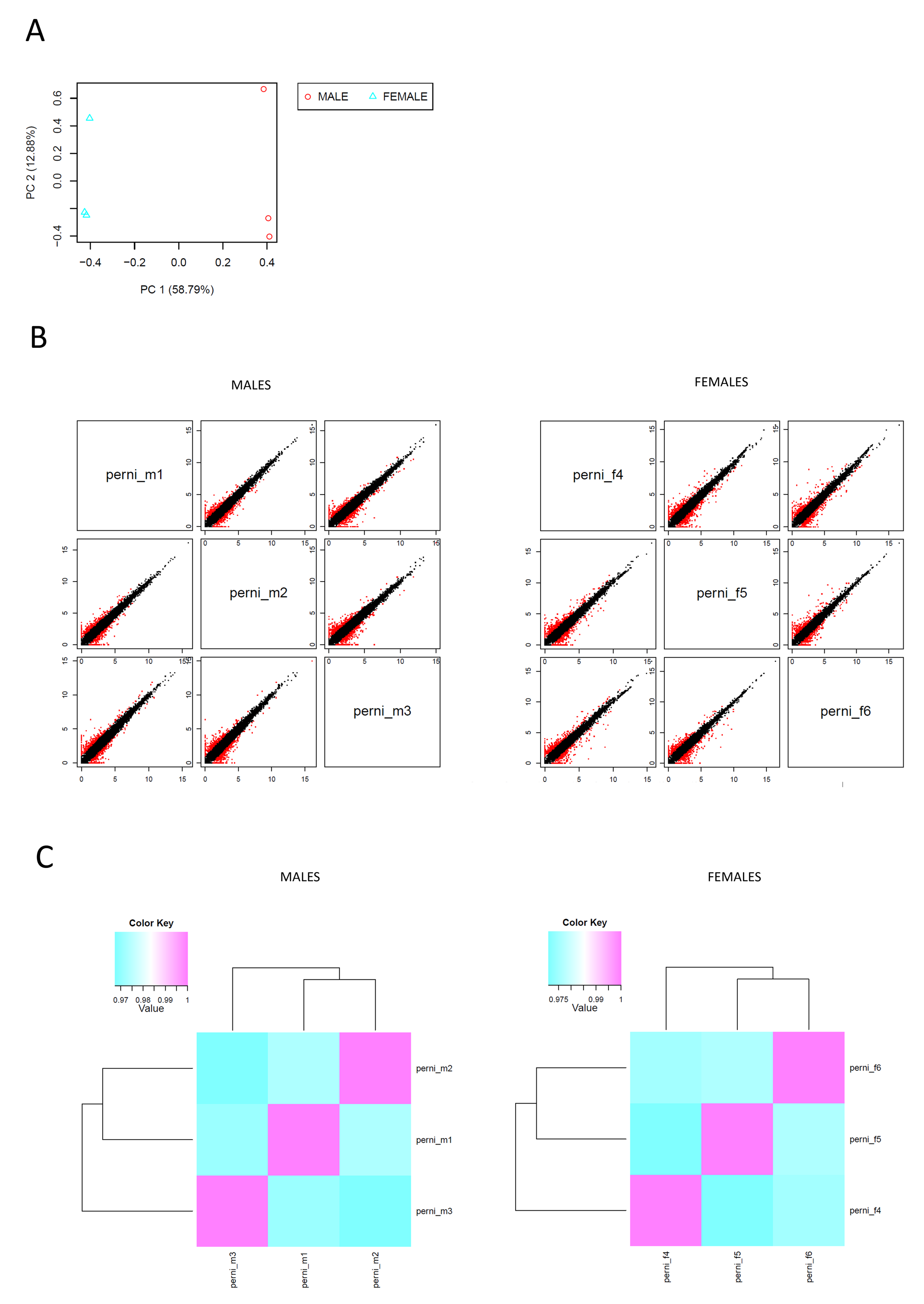

Supplement: Additional file 11: Figure S3. — Correlation between sequencing replicates. The correlation analysis between the male and female read replicates was performed using the normalized FPKM values. (A) Principal component analysis. (B) Scattered plot of log2 FPKM values between replicates. (C) Pearson correlation values between replicates. (TIFF 480 kb) [file 12864_2015_2088_MOESM11_ESM.tif]

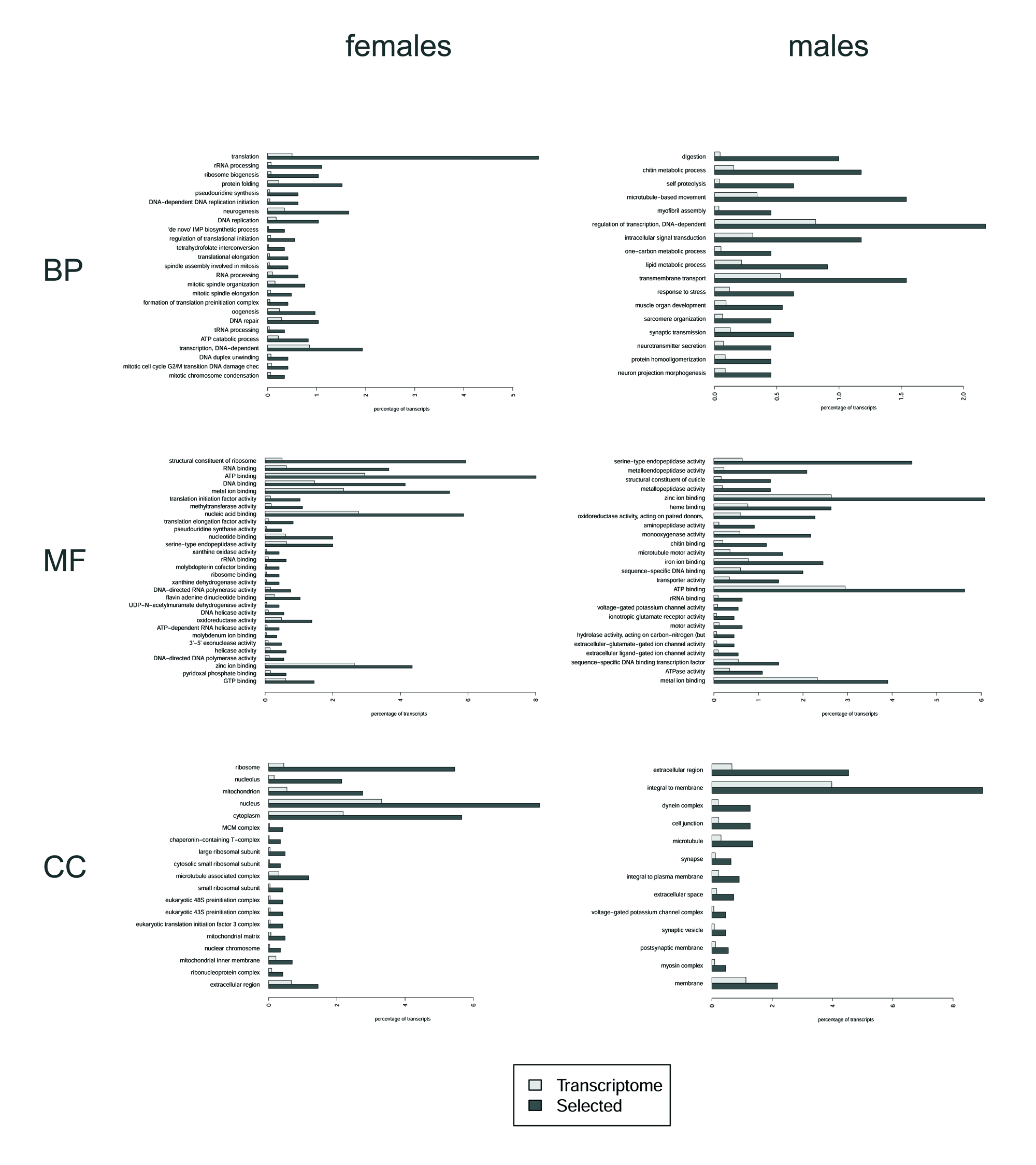

Supplement: Additional file 14: Figure S4. — Enriched GO term frequencies in males and females. Top GO enriched terms in female- and male-biased PERNI transcripts with FDR < 0.01 and FC > 2. BP (Biological Processes), MF (Molecular Function) and CC (Cellular Component). (TIFF 1289 kb) [file 12864_2015_2088_MOESM14_ESM.tif]
