# Supplementary material for: De novo assembly and sex-specific transcriptome profiling in the sand fly Phlebotomus perniciosus (Diptera, Phlebotominae), a major Old World vector of Leishmania infantum
Source: BMC Genomics. 2015 Oct 23;16:847. doi: 10.1186/s12864-015-2088-x (PMC4619268; doi:10.1186/s12864-015-2088-x)
Supplement: Additional file 16: Figure S5. — Sex-specific alternative splicing isoforms of the validated sex-biased transcripts. Clustal-W alignments of the sex-specific isoforms of the validated transcripts are reported. The 5’ and 3’ sequenced consensus sequences are represented in underlined case. Nucleotides matching with the sequenced consensus sequences are in bold case. (PDF 90 kb) [file 12864_2015_2088_MOESM16_ESM.pdf]

PpeFemale4 (Cluster-7890.0--c24385.g4.i1)

F4F GGTCCATATTTGCTCTTTATTCATTCACTCATCTTTTACATTATATTGAA
F4M GGTCCATATTTGCTCTTTATTCATTCACTCATCTTTTACATTATATTGAA

F4F AGACGTTCAACTTTATTTCTAATAAAATGCAGTTTTCTAAGATCTTCATC
F4M AGACGTTCAACTTTATTTCTAATAAAATGCAGTTTTCTAAGATCTTCATC

F4F TGGTCCATGTTCCTCTTCATCGTTTTTGCTTCGGTTGAAG----------
F4M TGGTCCATGTTCCTCTTCATCGTTTTTGCTTCGGTTGA**AGGTAAG**GTATT

F4F --CA
F4M TGATTTTAAATGTTTTGATAATTAAGGTAATTGTTTTATGTTAA**TCAGC**A

F4F AGTTCCAGTAAGTCAGCCAAATCCGAGCTCAGCCATCCAGTCCTAGGACC
F4M AGTTCCAGTAAGTCAGCCAAATCCGAGCTCAGCCATCCAGTCCTAGGACC

F4F CCGAAGACCACCATGTAATGAGGACCGATGCAAAAGGTTTTGCAGTCATT
F4M CCGAAGACCACCATGTAATGAGGACCGATGCAAAAGGTTTTGCAGTCATT

F4F TGAACCGTGAAGGAAAATGTATTAATAACAGGTGTTTCTGCTTGAAACGT
F4M TGAACCGTGAAGGAAAATGTATTAATAACAGGTGTTTCTGCTTGAAACGT

F4F AATAACCGGGATATGGCGAAGGTTAAACCAAACCATTCAGAATTGCCTGA
F4M AATAACCGGGATATGGCGAAGGTTAAACCAAACCATTCAGAATTGCCTGA

F4F AATGGTCAACGAGGCCCAGTAAATATGTAATTTAAATGACTTCATCGCTT
F4M AATGGTCAACGAGGCCCAGTAAATATGTAATTTAAATGACTTCATCGCTT

F4F GA
F4M GA

PpeMale5a (Cluster-10369.0--c19338.g1.i1)

M5aM	CAAATTTAATTTCCATACAAATATATATAGAGTCTCACTTGAACTGTTCT
M5aF	CAAATTTAATTTCCATACAAATATATATAGAGTCTCACTTGAACTGTTCT
M5aM	TTAGTTTCATGAAGAAATTGTGAAAATGAAGATCTTTCTATTGACTTTGT
M5aF	TTAGTTTCATGAAGAAATTGTGAAAATGAAGATCTTTCTATTGACTTTGT
M5aM	TTGCCCTCCTAATCATTGGAGGAATGGAAGGAACAGATGCGGTTGCTGAA
M5aF	TTGCCCTCCTAATCATTGGAGGAATGGAAGGAACAGATGCGGTTGCTGAA
M5aM	GGCGAATCGACGGGATGTATAATGTGTCCAGAGTCGTCTTTAGATGGTGG
M5aF	GGCGAATCGACGGGATGTATAATGTGTCCAGAGTCGTCTTTAGATGGTGG
M5aM	AAACGAAAATG---------------------------------------
M5aF	AAACGAAAAT**GGTAAG**AATTAAAAAAAAAATCTTTTTATTTGTTTGTGAA
M5aM	--
M5aF	AATTTTGATTATTTTTGTGCCTGATAAAAATTCCTGTACCGGGAATCGAA
M5aM	--
M5aF	CCCGGGGCATTTCAAATCCGATGAAAGGTTTTTAATACGTTCAACCCAGG
M5aM	--
M5aF	TTCGAATCCCAATAGATTTTTTCGGGTTTAATAATCGCCTGAACTAACTT
M5aM	--
M5aF	AAAAAAATATTTTTTATCTTAGAAGAAAAAGAATGAAACAGTTTTGGATG
M5aM	--
M5aF	ATGAAAGCGATGGTGGTGTGAATTTAAAATTTCTAACACTGTTTATTTTG
M5aM	---------------TTATGAGGACTCAAAGACATGTTCCAAATAATCCG
M5aF	TAATTTTCTTT**ACAGT**TATGAGGACTCAAAGACATGTTCCAAATAATCCG
M5aM	CCGGCACCTGCACCGGCTCCAAACCCATTGGATAAAGTGCAAATTTATCA
M5aF	CCGGCACCTGCACCGGCTCCAAACCCATTGGATAAAGTGCAAATTTATCA
M5aM	CGTTGGAAATGTTAGTAATACCTAATGTGATTTGACTTCCCGATCAAATA
M5aF	CGTTGGAAATGTTAGTAATACCTAATGTGATTTGACTTCCCGATCAAATA
M5aM	AAGGGGATGCCTTTAACAGTCGTCGTTAACATTTCCTACACAACAAGAAT
M5aF	AAGGGGATGCCTTTAACAGTCGTCGTTAACATTTCCTACACAACAAGAAT
M5aM	TATTTTGTTGTAAAACGAAAAATGAAAAAATTTTCATCAATAAATGCATT
M5aF	TATTTTGTTGTAAAACGAAAAATGAAAAAATTTTCATCAATAAATGCATT
M5aM	TAATGATGGAAAAAAAAAAAAA
M5aF	TAATGATGGAAAAAAAAAAAAA

PpeMale8 (Cluster-14619.0--c18223.g1.i1)

M8M CTTTCTAGTAGTTTGTCATCAAGTTCCAAAAGTGAATATGAATATTTTTG
M8F CTTTCTAGTAGTTTGTCATCAAGTTCCAAAAGTGAATATGAATATTTTTG

M8M CTGTAGGAATATTCTTATTGATGTTATTCAGAATAGGAGGATGCTCGAAT
M8F CTGTAGGAATATTCTTATTGATGTTATTCAGAATAGGAGGATGCTCGAAT

M8M CTAATCAGAG--
M8F CTAATCAG**AGGTA**G**A**TATAAGATAATAAACTAATAATTCTTGATGGACGT

M8M --------------------ATCAATCACGCTTTACCAGACAAGATTTTT
M8F CTAACAGCCTTTTATT**TCAGA**TCAATCACGCTTTACCAGACAAGATTTTT

M8M TTACCTCTGCAGTTCAACCACCTGACTATGATGATGGAACAGGAGGTCCA
M8F TTACCTCTGCAGTTCAACCACCTGACTATGATGATGGAACAGGAGGTCCA

M8M AATGTGTATTATTCAGTACCAGATGGCACTGAAATGCAGCCTTCACCAAT
M8F AATGTGTATTATTCAGTACCAGATGGCACTGAAATGCAGCCTTCACCAAT

M8M GATGCAACCAAGACGACAAGAGCAGCAATATCATCCATATGAAACGCCTT
M8F GATGCAACCAAGACGACAAGAGCAGCAATATCATCCATATGAAACGCCTT

M8M ATCCAGATGATATTAGGCAACCACCTTCATATGAATTTTTGCCACCCCCC
M8F ATCCAGATGATATTAGGCAACCACCTTCATATGAATTTTTGCCACCCCCC

M8M AGTGGTTGAAAATATTCGATCATTTTGAATGTTTTACCACCAATAAAAAA
M8F AGTGGTTGAAAATATTCGATCATTTTGAATGTTTTACCACCAATAAAAAA

M8M GACAAAATAAAAAAGACGAAATCTCGACCGTTCGTAACTCCAGGGGTCCA
M8F GACAAAATAAAAAAGACGAAATCTCGACCGTTCGTAACTCCAGGGGTCCA

M8M AAA
M8F AAA

PpeMale15 (Cluster-18862.0_c3474_g1_i1)

M15M AATAAACTTGTGATCGATTGATCTTTAAACATTGTGAAAATGAATTTTCA
M15F AATAAACTTGTGATCGATTGATCTTTAAACATTGTGAAAATGAATTTTCA

M15M TCTAGTACTAGCAGGAGTTTTCTTAGTGATGTTCTTTGGATCAG------
M15F TCTAGTACTAGCAGGAGTTTTCTTAGTGATGTTCTTTGGATC**AGGTAA**AC

M15M --
M15F TATTAGATTCAATAATAATTCCATTTCCCACTTAATATCCTGTCAAATAC

M15M --------GACATTGCTCAGATGAACTTCGAGATGAATCCCGTCTTCCCA
M15F TTAT**CCAGG**ACATTGCTCAGATGAACTTCGAGATGAATCCCGTCTTCCCA

M15M GATCTGCTTTTGCATCCCCACAAGATTATGGACAATATCCGGAAGTATAT
M15F GATCTGCTTTTGCATCCCCACAAGATTATGGACAATATCCGGAAGTATAT

M15M GCACAATATTACCCGGCCGTAGATCCACAAAATCCGGGATATACCATTCA
M15F GCACAATATTACCCGGCCGTAGATCCACAAAATCCGGGATATACCATTCA

M15M GCCTGACCCCCCGCCGCCACAGTACGGTCATGGTATTTATCAATACGATT
M15F GCCTGACCCCCCGCCGCCACAGTACGGTCATGGTATTTATCAATACGATT

M15M ACCCACTGAAAGGTCGTTGGAGGAAGTGAAAGAATCTGATTGAGAAATTG
M15F ACCCACTGAAAGGTCGTTGGAGGAAGTGAAAGAATCTGATTGAGAAATTG

M15M AAAATCTGAGATTCTTATCCAGGATTTTGGACCTTTGCGGAATAATATTT
M15F AAAATCTGAGATTCTTATCCAGGATTTTGGACCTTTGCGGAATAATATTT

M15M CTTTTCTGCACGAAAAATAATTCAGCAAATAAAGGAAACTTCAAACATTA
M15F CTTTTCTGCACGAAAAATAATTCAGCAAATAAAGGAAACTTCAAACATTA

M15M AAAAAAAAAAA
M15F AAAAAAAAAAA

PpeMale17 (Cluster-19669.0--c22663.g1.i6)

M17M	CAACCAGAATTGTACAATAAGTATAACTAAACCAAAACGCAGCATGAAAT
M17F	CAACCAGAATTGTACAATAAGTATAACTAAACCAAAACGCAGCATGAAAT
M17M	ATATTTTCATACTGACCATTTTTCAAATTATTTTCACAAAATACATATAC
M17F	ATATTTTCATACTGACCATTTTTCAAATTATTTTCACAAAATACATATAC
M17M	CTAATATAAGTCTGATATGTCTTTAAGACATACAAAATTCTTCAATTTTG
M17F	CTAATATAAGTCTGATATGTCTTTAAGACATACAAAATTCTTCAATTTTG
M17M	CTTGCTCAGTGAGAAAAGCTCTTATAATGTGTAGAATAGTTTTGGCAACA
M17F	CTTGCTCAGTGAGAAAAGCTCTTATAATGTGTAGAATAGTTTTGGCAACA
M17M	TCTACGAAATGGATTTCGCGAAGTGCATACCGACTAGACCTGGATCCATT
M17F	TCTACGAAATGGATTTCGCGAAGTGCATACCGACTAGACCTGGATCCATT
M17M	CCTTAACGAACTACACAATATATTTCATAAAGACCAATTAACCGCCTGAT
M17F	CCTTAACGAACTACACAATATATTTCATAAAGACCAATTAACCGCCTGAT
M17M	TTCATGTAATTACAAATTCAATTAAAAAATTGATAAAACCTAATGTGAAT
M17F	TTCATGTAATTACAAATTCAATTAAAAAATTGATAAAACCTAATGTGAAT
M17M	TTCTAGTATAAAAATACTTCTAGGAGTACAAACTCAAGTGATTTTCACAC
M17F	TTCTAGTATAAAAATACTTCTAGGAGTACAAACTCAAGTGATTTTCACAC
M17M	AAATTTTCACTAGATTTTGACTAATGGTTGGTACATTCAAGAGTTTCAAC
M17F	AAATTTTCACTAGATTTTGACTAATGGTTGGTACATTCAAGAGTTTCAAC
M17M	TGAAGTCAAAATGAAAGGTCTTTTAGCAGTGATTCTGTTGATTAACATTT
M17F	TGAAGTCAAAATGAAAGGTCTTTTAGCAGTGATTCTGTTGATTAACATTT
M17M	TGGGACAAA---
M17F	TGGGACA**AAGTAAGT**TCAAAATATTCACAAATAAACAATATTCTTTTACC
M17M	------------------------------GTCAATGCGAGCCTCGAGAT
M17F	TATAATAATATTTTATTTATATTAAA**TCAGG**TCAATGCGAGCCTCGAGAT
M17M	TGGCCACATGTTAGTCAACAATCCTCGTCCCAAAACAGGATTGACAGTTC
M17F	TGGCCACATGTTAGTCAACAATCCTCGTCCCAAAACAGGATTGACAGTTC
M17M	ATTCGTTGGATGTGGTCCTGATTGTGAAAATCGTCCACCACCAGGTGGTA
M17F	ATTCGTTGGATGTGGTCCTGATTGTGAAAATCGTCCACCACCAGGTGGTA
M17M	GGCCGCGGCCACCACCAGGTGGTAGACCGCGGCCACCACCAGGTGGTTAT
M17F	GGCCGCGGCCACCACCAGGTGGTAGACCGCGGCCACCACCAGGTGGTTAT
M17M	CCGTGGCCACCCTCACCACCAACTCCACCAAGACCACCACTAGGACAACC
M17F	CCGTGGCCACCCTCACCACCAACTCCACCAAGACCACCACTAGGACAACC
M17M	AGCATGCTATCCAGCATGCTGTGTGCTTATGCCATGCTATCCCGGCTAAC
M17F	AGCATGCTATCCAGCATGCTGTGTGCTTATGCCATGCTATCCCGGCTAAC
M17M	CGTTATTAATCCAAATTGTTAAAGAAAATTTATTTCAAAATAAAGAAAGA
M17F	CGTTATTAATCCAAATTGTTAAAGAAAATTTATTTCAAAATAAAGAAAGA
M17M	TTCCCCGAAAAGCTGATCTGCAAAATTCCTAAATAAAAAAAAAAAA
M17F	TTCCCCGAAAAGCTGATCTGCAAAATTCCTAAATAAAAAAAAAAAA
