# Supplementary material for: De novo assembly and sex-specific transcriptome profiling in the sand fly Phlebotomus perniciosus (Diptera, Phlebotominae), a major Old World vector of Leishmania infantum
Source: BMC Genomics. 2015 Oct 23;16:847. doi: 10.1186/s12864-015-2088-x (PMC4619268; doi:10.1186/s12864-015-2088-x)
Supplement: Additional file 18: Methods S1. — List of primers utilized in the present paper. (PDF 120 kb) [file 12864_2015_2088_MOESM18_ESM.pdf]

Methods S1

List of primers utilized in the present paper

Real Time PCR

Normalizator genes

qP119c+: 5'- GGAGGCTGCCTTCTATC-3'
qP119c-: 5'- GAATTGGTTGCCTCTGCTG-3'
qGAPDH+: 5'- AGTCCACGGGAGTGTTTACCA-3'
qGAPDH-: 5'- CGCCGCCCTTGAGATG-3'
qGPDH+: 5'- TTCCAAAGACGCCGACATC-3'
qGPDH-: 5'- GGCCAAGCCACGAATGAA-3'
qP450+: 5'- CCCAAGGGAATGCCTGTTTA-3'
qP450-: 5'- AGATTTTCGGATCACGATGGA-3'
qSOD+: 5'- TTATCCAATTCCTGCGTTCCA-3'
qSOD-: 5'- GGCAAATGCCAGACAGTCAA-3'

unbiased genes

qUnbiased1+: 5'-+ TGGCTCTGATGGCGAGATC-3'
qUnbiased1-: 5'- GGACGCTGGAAAGATTGCT-3'
qUnbiased2+: 5'-GACCTGCTGTGCTCATGCA-3'
qUnbiased2-: 5'- CTGTCTTCTCGTCGCGTGAA-3'
qUnbiased3+: 5'- CAGCTTGCGAGGGAAGA-3'
qUnbiased3-: 5'- CCACCAGATAAGAGAGTCA-3'
qUnbiased4+: 5'- GACATTGCGTGGCAACACA-3'
qUnbiased4-: 5'- AGGGATACTTTGGGCAGTTCAG-3'

Female-biased genes

qFemaleBiased1+: 5'- CACCGTTTGTCTGGATCACAA-3'
qFemaleBiased1-: 5'- TCTGATTCCTTCTGGCGATGA-3'
qFemaleBiased2+: 5'- TCCGGGAAGGTCAGATTGG-3'
qFemaleBiased2-: 5'- GCTGCGGCGGCTTTAGT-3'
qFemaleBiased3+: 5'- GGGCAACAGTTAAGGGTGACA-3'
qFemaleBiased3-: 5'- GCGTCCAGAAAGGCCATTC-3'

Male-biased genes
qMaleBiased1+: 5'- GCATCCACGAGACCGTCTACA-3'
qMaleBiased1-: 5'- CATACAAGTCCTTGCGGATGTC-3'
qMaleBiased2+: 5'- CAAGCCGCGAAGTTCGA-3'
qMaleBiased2-: 5'- TCGCACGATCCCCCTACTAG-3'
qMaleBiased3+: 5'- ATGTTCCACATCCAACTGATCCT-3'
qMaleBiased3-: 5'- GAAGAAACTCCCGGGTCATG-3'

Semi-quantitative PCR

Normalizator and positive control genes

PpeP119+: 5'-ACCGTCACCATTGCAGAAAG-3'
PpeP119-: 5'-ATTCAGGTCCCTGTCTTCTAAGT-3'
PpeCytP450+: 5'-CTACTACGAATCCGCAAACC-3'
PpeCytP450-: 5'-ATTAGTCGATTCAGGGAATAC-3'
PpeSOD+: 5'-AGGAGGCGTCGTTCGTGAAT-3'
PpeSOD-: 5'-CCTTGCCATGAGGATTGTAG-3'
PpeGAPDH1+: 5'-GAATGGTCAGCAGATCACTG-3'
PpeGAPDH1-: 5'-GGGGATGATATTCTGCTGAG-3'
PpeApy+: 5'-CAAGTTGATTGTCGGATCAG-3'
PpeApy-: 5'-TTGATGTCAATCACTGTGCC-3'

PpeAct3+: 5'-AGATGTGTGATGAAGATGCG-3'
PpeAct3-: 5'-CCAGAGACGTGGAGGCGGCC-3'

Female-biased genes

Ppe_Female1F: 5'-AACACGATGCCGATTGCCTG-3'
Ppe_Female1R: 5'-ACGGTGTTCTTAGCTCTGCC-3'
Ppe_Female2F: 5'-GTCCATTCAGCAGGGAAGAC-3'
Ppe_Female2R: 5'-GAAGAAGTTATCCTGCGAGTG-3'
Ppe_Female3F: 5'-TTCCAAGCACCCAAGATGAC -3'
Ppe_Female3R: 5'-TCCGCCGGTTAGAAGAGGTT -3'
Ppe_Female4F: 5'-ATCTTCATCTGGTCCATGTTC-3'
Ppe_Female4R: 5'-CCTCGTTGACCATTTCAGGC-3'
Ppe_Female5F: 5'-GCCTTAGCTTCTACTACTCC-3'
Ppe_Female5R: 5'-GGGACAGAATAGATGACTGG-3'
Ppe_Female6F: 5'-CAATGTTCAACAATCGCTCAG-3'
Ppe_Female6R: 5'-ATGAGTCAGCTTGTGAAACTC-3'
Ppe_Female7F: 5'-AGTTGCATACTTCAGGGAAG-3'
Ppe_Female7R: 5'-GGTGTTGTGGAGACTACCG-3'
Ppe_Female8F: 5'-TGTTCCAGACCGACATCAG-3'
Ppe_Female8R: 5'-TGAACTGGGCATAGAAATCG-3'
Ppe_Female9F: 5'-GGACGAAAGGTGAAATCTGA-3'
Ppe_Female9R: 5'-CTCCGCACGAAGTAGAAG-3'
Ppe_Female10F: 5'-GTGTAGGATAGAGTACCAGA-3'
Ppe_Female10R: 5'-TAGAGCATCAAGGCATCACC-3'
Ppe_Female11F: 5'-GAAGAACATCGAGGAGATAC-3'
Ppe_Female11R: 5'-ACGTCTGTTGTGTTTGTCTC-3'

Male-biased genes

Ppe_Male1F: 5'-GGACTCCAAGGAGCACCATA-3'
Ppe_Male1R: 5'-GTGTTTAATGTCCGTCTGTG-3'
Ppe_Male2F: 5'-GTGTCTGTTGTAATAGCTCG-3'
Ppe_Male2R: 5'-AAGCTTAATCAGAGTATCCC-3'
Ppe_Male3F: 5'-GAAAGGAGAACAGTTTTGTG-3'
Ppe_Male3R: 5'-GAAAATGTTGCTCTATGTCTAC-3'
Ppe_Male4F: 5'-GCGTTTGCCATCTTGGCCTT-3'
Ppe_Male4R: 5'-TTTTCCATTGATAACAGGGGC-3'
Ppe_Male5AF: 5'-ATTGACTTTGTTTGCCCTCC-3'
Ppe_Male5AR: 5'-GGAAATGTTAACGACGACTG-3'
Ppe_Male5BF: 5'-GATGCGATTGATGAAGTCAG -3'
Ppe_Male5BR: 5'-AACCAATTCTTGTTGTGTGG -3'
Ppe_Male6F: 5'-GAATGTGCTAGTGCTTCTCC-3'
Ppe_Male6R: 5'-ATATTGGTCATCCCGCCAC-3'
Ppe_Male7F: 5'-GGAGGCTATAGAAGAGGATAG-3'
Ppe_Male7R: 5'-CTTCTTCAGGCTCTAATGCG-3'
Ppe_Male8F: 5'-TCAGAATAGGAGGATGCTCG-3'
Ppe_Male8R: 5'-ATGATATTGCTGCTCTTGTC-3'
Ppe_Male9F: 5'-ACAGTCTCAACAGTCTCAAC-3'
Ppe_Male9R: 5'-ATTTCAATGTGACCGATGCC-3'
Ppe_Male10F: 5'-TGCTCAACCACCTTCTCCAG-3'
Ppe_Male10R: 5'-ATCCAAAGTATCAACGACGG-3'
Ppe_Male11F: 5'-ATTCTTAGTCTTGTTCTTCGG-3'
Ppe_Male11R: 5'-ATGTGCTTGTTTTGAGGTCC-3'
Ppe_Male12F: 5'-ATGTTATGACTGCGCTCCTC-3'
Ppe_Male12R: 5'-TCGTCATCACGGTCGTCAC-3'
Ppe_Male13F: 5'-GGCATGAATGAACTTCAAGG-3'
Ppe_Male13R: 5'-GTTTCTAAGGCTTCAACGAC-3'
Ppe_Male14F: 5'-CAACTACCACCATATTCCG-3'
Ppe_Male14R: 5'-TCCGGTTAACTGAAATAGAAG-3'
Ppe_Male15F: 5'-GTGATGTTCTTTGGATCAGG-3'
Ppe_Male15R: 5'-TTCTTTCACTTCCTCCAACG-3'
Ppe_Male16F: 5'-GATAAAACCTCTCCTTGAAGC-3'

Ppe_Male16R: 5′-CATTTGTACGGATGTCATTCC-3′
Ppe_Male17F: 5′-AAGTGCATACCGACTAGACC-3′
Ppe_Male17R: 5′-AGGACCACATCCAACGAATG-3′
Ppe_Male18F: 5′-CAAAGTTGACTGTCGAAGG-3′
Ppe_Male18R: 5′-ATCAGAGTGATTTGGGTTCG-3′
Ppe_Male19F: 5′-GTGTCAGTTGAATTACGAGG-3′
Ppe_Male19R: 5′-CGATCCAACATCACACGAAG-3′
